# Nanocomposites Based on Thermoplastic Polymers and Functional Nanofiller for Sensor Applications

**DOI:** 10.3390/ma8063377

**Published:** 2015-06-10

**Authors:** Serena Coiai, Elisa Passaglia, Andrea Pucci, Giacomo Ruggeri

**Affiliations:** 1 Istituto di Chimica dei Composti Organo Metallici (ICCOM), Consiglio Nazionale delle Ricerche, UOS Pisa, Via G. Moruzzi 1, Pisa 56124, Italy; E-Mail: coiai@pi.iccom.cnr.it; 2 Dipartimento di Chimica e Chimica Industriale, Università di Pisa, Moruzzi 13, Pisa 56124, Italy; E-Mails: andrea.pucci@unipi.it (A.P.); giacomo.ruggeri@unipi.it (G.R.)

**Keywords:** cationic and anionic clays, photoresponsive properties, thermoplastic polymers, nanocomposites, noble metal nanoparticles, carbon nanotubes, polymer sensor

## Abstract

Thermoplastic polymers like polyolefins, polyesters, polyamide, and styrene polymers are the most representative commodity plastics thanks to their cost-efficient manufacturing processes, excellent thermomechanical properties and their good environmental compatibility, including easy recycling. In the last few decades much effort has been devoted worldwide to extend the applications of such materials by conferring on them new properties through mixing and blending with different additives. In this latter context, nanocomposites have recently offered new exciting possibilities. This review discusses the successful use of nanostructured dispersed substrates in designing new stimuli-responsive nanocomposites; in particular, it provides an updated description of the synthetic routes to prepare nanostructured systems having the typical properties of thermoplastic polymers (continuous matrix), but showing enhanced optical, conductive, and thermal features dependent on the dispersion topology. The controlled nanodispersion of functional labeled clays, noble metal nanoparticles and carbon nanotubes is here evidenced to play a key role in producing hybrid thermoplastic materials that have been used in the design of devices, such as NLO devices, chemiresistors, temperature and deformation sensors.

## 1. Introduction

Polymer nanocomposites are considered the materials of the 21st century. They combine the use of a nanostructured inorganic or organic filler with size typically of 1–100 Å in at least one dimension, and a polymeric continuous matrix. Their advantage over conventional composite materials founds on the extremely high surface area of the fillers, which have proportionally more surface atoms than their microscale counterparts, thus allowing intimate interphase interactions and conferring extraordinary properties to the polymer. The nanosize favours the use of smaller amounts of fillers and a more effective transfer to the polymer matrix of their unique molecular properties. Notably, in the nanoscale range, materials may present different opto-electronic properties, which in turn affects their optical, catalytic and other chemical properties, thus suggesting applications in the field of functional materials, such as, temperature sensors, linear polarizers, optoelectronic and chemiresistor devices. Among the polymeric matrices used in the preparation of nanocomposites, thermoplastic polymers represent a class of interest both for scientific research and application at industrial level. Polymers such as polyolefins, polyesters, polyamides, homopolymers and copolymers of styrene, are known for their good mechanical properties, durability and versatility in processing that allow their use in many of the different forms used in the sensing devices of interest in this review.

Sensing is what we can define as the property of a traditional device to detect events and provide a corresponding output, generally as an electrical or optical signal. A “good sensor”, even nanostructured, has to provide a fast change (in structure, shape, optical response, conductivity as examples of “probe property”) under stimuli of its environment, by coming back to the pristine state in short time and by completely recovering the starting energy level.

Many reviews have been focused onto the preparation and the characterization of nanocomposites from thermoplastic polymers often addressed to highlight their improved thermal and mechanical features when compared to polymer matrix or traditional composites [1,2,3,4,5,6].

No specific article is reporting an exhaustive overview of scientific literature on nanocomposites for sensor applications even if these devices have attracted considerable attention due to their interesting opto-electronic properties, high surface area, and good environmental stability provided by the polymeric continuous phase. In addition, optically- and electronically-conductive polymer nanocomposites have good sensitivity and reproducibility to various external stimuli, and fast response time.

The use of functional nanofiller in the design of nanocomposite sensors from thermoplastic matrices can combine the peculiar stimuli-responsive features of labeled nano(hybrid) systems with the generally improved characteristics of thermoplastic-based nanocomposites. To optimize the sensor characteristics it necessary that the probe property (optical features or conducting characteristics) is better preserved or even improved by the efficient (nano)dispersion of the functional filler within the thermoplastic matrix. When referring to the preparation procedure, one should keep in mind the two main distinct routes available to nanocomposite preparation: The “top-down” approach and the “bottom-up” approach. The most sustainable process for nanocomposites preparation starting from thermoplastic polymers is certainly that based on the reactive formation of a nanophase during blending with a polymer. The exfoliation of layered silicates, or minerals, during mixing with the polymer, allows the dispersion at the nanoscale being polymer/inorganic material interfacial interactions and thermomechanical stress induced by the machine, both effective in the filler transformation into nanoparticles. This approach is not easily applicable and transferred to the preparation of nanocomposites for sensing based onto nanolayered systems. The different aspects affecting the design of these hybrids will be then examined through inspection of compelling literature’s examples. Polymerization reactor blending can give exfoliation because of the improved monomer/clay interactions and polymerization energy evolution. Also, this preparation route will be investigated for the preparation of chromogenic nanocomposite sensors.

Moreover, the formation of noble metal nanoparticles through a chemical reaction performed in the presence of a polymer or the blending of preformed stable nanoparticles with a polymer will be examined and relevant examples for applications as sensors will be described in detail. Finally, carbon nanotubes, which possess substantial electrical properties, and, in particular, their incorporation into thermoplastic polymers will be considered as new functional materials for sensing external solicitations such as temperature and mechanical stress as well as exposure to vapours of volatile organic compounds.

The review is divided into four sections, depending on the nature of nanofiller (cationic clays, anionic clays, noble metal nanoparticles, carbon nanotubes). They will address the preparation, the inherent properties, the possible functionalization, as well as the methodology of dispersion in the polymer matrix and eventually some of the more recent examples of application as functional materials for sensor applications.

In order to provide the review with better value and adequately criticized affordable information, the examples selected fall in the area of authors’ expertise and research activities.

Following these general lines, this review will report a substantial number of examples we arbitrarily considered suitable to provide the reader with illuminating information about the main topic, disregarding a full coverage which would be impossible for space limitations and merely informative rather than formative and impressive. As mentioned above, biomedical applications are not considered.

## 2. Cationic Clay Thermoplastic Polymer Sensors

### 2.1. General Introduction

Cationic clay minerals, such as smectite, are characterized by a layered structure due to the condensation space of an octahedral (O) Al_2_O_3_ or MgO planar sheet between two planar tetrahedral (T) SiO_2_ sheets, generating a TOT layer characterized by a thickness of a nanometer. Isomorphic substitutions with metals of lower valence generate negative charges compensated by inorganic exchangeable cations, which are located on the surface of TOT layers [7,8] These exchanged cations by interacting with the negative charge delocalized onto the surface of platelets, induce the stacking of TOT platelets thus giving rise to a sandwich structure of piled lamellae (the clay tactoid structure).

Over the past decades, nanocomposites obtained by dispersion of these clays in polymer matrices have attracted great interest, both in academia and in industry, owing to the capability of such kind of fillers (if dispersed at the nanoscale) to impart to the resulting materials remarkable improvements of properties in comparison with the starting polymer matrices or conventional micro-composites. These enhanced features generally include mechanical, and thermal performances, heat resistance, flammability, gas permeability reduction, solvent and chemical resistance. These features are the result of an inorganic/organic co-continuous phase formation (hybrid system). Notably, its volume fraction in the composites is maximized by the dispersion at the nanoscale through the intercalation of macromolecules within the interlayer space. Therefore, the final properties are tailored as if the composite system would be totally interfaced. Moreover, the cationic clays can accommodate a huge variety of guest molecules due to their cationic exchange capacity thus also providing expanded interlayer spaces, while maintaining unalterable the piled structure. The related composites may also show additional specific properties if the filler (clay) bears organic species having magnetic, non linear optics, biological or pharmaceutical activities [9,10,11].

A wide range of approaches to modify clay is reported in literature [12]; among them, the most common procedure to adsorb ionic functional surfactants onto clay mineral particles is the cation exchange. Surfactant cations can modify the nature of the clay mineral particles from hydrophilic to hydrophobic, necessary to be dispersed in apolar matrices as most of the thermoplastic commodities. In addition if the intercalant bears a specific functionality (for example a cationic photoreponsive dye) the related property (optical property) can be tentatively transferred to the polymer matrix by simply dispersing the functional clay in the polymer itself. In this sense hybrid organo-inorganic systems bearing encapsulated/intercalated photoactive (photochromic) molecules have been investigated in order to create new templates for optical and electronic devices. The immobilization of photoactive guest molecules with a preferential arrangement in organized nanostructured inorganic stacked platelets can lead to macroscopic alignment of photofunctional molecules. This architecture may open promising and interesting features for applications in nonlinear optics (NLO) or to aggregation of the absorbed dyes reducing the photoresponsivity of dye/clay systems. The host-guest and guest-guest reciprocal-mutual interactions play an important role in the activated clay preparation and in the following design of polymer-based composite devices. A really impressive number of studies have been carried out to investigate the possibility to adsorb/intercalate dyes onto/into cationic clays, by deepening insight the effects of dye-dye and dye-clay interactions onto the final optical properties in comparison with the behavior of the same dyes in solvent solutions. However, to date, a few studies report significant advances in the use of these devices for the preparation of sensors in thermoplastic polymer matrix. Those results mainly concern the evaluation of the morphological features and the transfer of the dye optical properties to polymer bulk.

### 2.2. Preparation of Photoactive Cationic Clays

Photoresponsive molecules with different optical properties (photoactive and/or photochromic behavior) have been embedded in cationic clays to improve their photo-, thermo- and chemical stability. This incorporation aimed at the modification of their electrical, magnetic and optical properties for the design of probes in the characterization of the solid constrained nanostructures and as functional fillers in polymer nanocomposites after dispersion. In the frame of the simple photoactive molecules and by referring to the recent literature, the most studied dyes are those having fluorescent features with particular reference to derivatives of rhodamine, anthracene, perylene, and other condensed aromatic compounds (Nile blue, Methylene blue only as examples). All these dyes bear a photoactive chromophore and a cationic group necessary for the ionic exchange. Azobenzene dyes as well as spiropyran derivatives, even designed in dendritic intercalated structures, account mostly to photochromic behavior for the development of second-order NLO materials. Also the easy preparation of nanopigments has been recently reported by intercalating dyes in the modified and unmodified clays [13,14].

The optical properties are environmentally sensitive and, according to the theory of exciton splitting, (see for example the review of F. Lopez for the rhodamine dye intercalated in clays [15]), depend on the arrangements and spatial configuration of chromophores undertaken in the confined interlamellae spacing. Aggregates play a fundamental role in the fluorescence characteristics of the dye molecules by decreasing, in some cases, the fluorescence quantum yield. In general, there are two main types of dye aggregates, H- and J-aggregates. The H-aggregates are characterized by sandwich type of a structure and absorb light at the higher energies than the isolated dye cations. These aggregates become non-fluorescent species, because of the very fast and non-radiative internal conversion from the spectroscopically active highest excited state to the lowest excited state, which is not fluorescent. The J-aggregates with a head-to-tail intermolecular structure absorb light at lower energies than the corresponding dye monomers; the allowed transition is that involving the ground and the lowest excited state; these aggregates are generally fluorescent and a bathochromic band (J-band) with respect to the monomer is observed in both absorption and fluorescence spectra. The crucial problem to be solved in a preparation of hybrid inorganic–organic systems with good photoresponsive properties is then the suppression of H-aggregates formation.

In the presence of ionisable groups, such as carboxylic acids or ammine groups, the distribution of the charge in heteroaromatic skeleton [16,17] but overall the hydrophobic/hydrophilic character of the photoactive groups and the inorganic substrates have been extensively studied as affecting the extent of quencher aggregates formation. Taking into account that the aggregation of rhodamine (as example) in liquid solution is drastically reduced in hydrophobic media, the incorporation of rhodamine in organophilic clays has been investigated as a good strategy for reducing its aggregation. Sasai *et al.* [18,19,20] prepared highly luminescent films of clay minerals with rhodamine 6G, where the molecular aggregation of the dye was suppressed by a premodification of the inorganic host with appropriate amounts of long-chain alkylammonium ions. This represents one of the most common strategies to enhance the interfacial properties and thus the dispersion level of the nanofiller in apolar thermoplastic commodities. In this regard, one of the main goals of this approach is to find optimum conditions to impart targeted light-responsive properties to commercially available organo-clay mineralsfor monitoring the morphology of polymer clay nanocomposites (profiting tools for real-time polymer flow visualization) [21].

Very interesting results have been obtained in this field by Salleres *et al.* [22,23] and Esposito *et al.* [24] both research groups working on organophilic smectite and different dyes. The incorporation of rhodamine 6G (R6G) in laponite clay, previously modified with dodecyltrimethyammonium chloride, produces hybrids with improved photophysical properties with respect to those obtained by using the unmodified clay.

As previously observed in ethanol solutions, the organic surrounding in organoclay films increases the fluorescence efficiency and lifetime, indicating a lower nonradiative deactivation from the monomer fluorescent excited state and, therefore, enhancing the photophysical behaviour of the dye, by reducing its tendency to aggregate.

**Figure 1:**
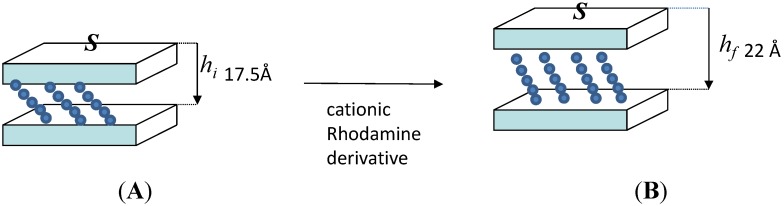
Possible arrangement of montmorillonite layers before (**A**) and after (**B**) the intercalation of rhodamine derivative. Reproduced from Esposito *et al.* [24].

These results are better justified by the accurate study of Esposito *et al.* [24] reporting the preparation of a photofunctional organo-montmorrillonite starting from a commercially available montmorillonite (Cloisite 30B, modified with methyl tallowyl bis-hydroxyethyl ammonium salt, MT2EtOH) that was functionalized by three fluorescent dyes: 9-anthracene ethanol, Nile blue A Perchlorate, R6G Perchlorate. Only the dyes containing the cationic species resulted intercalated and the best results in terms of intercalation efficiency, thermal stability and photophysical properties were obtained by using the Rhodamine derivative. It was observed that the presence of MT2EtOH moieties limit the tendency of the dye to aggregate, by increasing their angle of tilt up to 49°, allocating the adsorbed dyes molecules in the interlayer space in the form of monomers and/or fluorescent J-aggregates (Figure 1).

The structural stability of the modified clay seems to play a significant role in the following dispersion/embedding of the dye; with this aim recently Czìmerovà *et al.* [25] reported the results of R6G adsorption onto clay previously modified with different content of a polycation (poly(diallyldimethyl ammonium chloride), PDDA).

R6G monomers are well-known to show fluorescence at around 550 nm. The emission spectra in the presence of unmodified clay (Na-KF) suspension, without PDDA molecules are very similar to those with low content of PDDAThis implies that low amount of PDDA has only a marginal influence on the luminescence properties of these hybrid systems. Besides the low emission, a band at higher energies (at about 595 nm) can be observed. This band is attributed to the fluorescent J-bands of R6G dimers and higher-order aggregates (as already observed by Salleres *et al.* [23]).

The fluorescence intensity of monomers gradually increases with the amount of co-intercalated PDDA molecules. The high fluorescence intensity clarifies that the interaction between the intercalated R6G cations is drastically reduced in presence of the higher amount of co-intercalated PDDA molecules. It follows that the presence of polycations enhances the fluorescence capability and no fluorescence quenching is observed.

If the rhodamine dyes species require a co-intercalation process to improve their photophysical properties in constrained clay lamellae interspace, quite interesting results have been instead obtained by the intercalation of cationic perylene chromophore guest molecules in native un-modified MMT host [26]. The accurate characterizations performed onto the hybrids prove the arrangements of perylene molecules as monolayer that are tilted within the gallery by 24°–29° angle and assemble in J-type aggregation. This assembly allowed functional clays with excellent thermo- and photo-stability, even if to be improved with respect to the chromophore optical characteristics. There is no comparison with the sole rhodamine dyes species, but the absence of polar functionalities with the exception of cationic group necessary for anchoring to clay platelets can affect the formation of aggregates with different optical response.

Photochromic molecules have been attracted much attention due to their possible application as switching components; photochromism is, in fact, a reversible transformation of a single chemical species induced mostly by electromagnetic radiation. During photoisomerization the two isomers differ each other not only in their optical response, but also in their geometrical structures, redox potential, refractive indices and dielectric constants.

**Figure 2:**
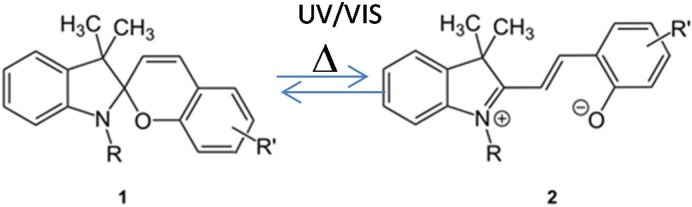
Structural changes of spiropyran during photochromism. Reprinted with permission from Kinashi *et al.* [27].

As an example, spiropyran derivatives are thermally reversible photochromic chromophores. UV light illumination induces photoisomerization from the colourless spiro (SP, 1) form to the colored merocyanine (MC, 2) form, and subsequent illumination with visible light results in a colour fading (Figure 2).

In addition, when placed in the dark, the MC form reverts to the SP form thermally. This kind of photoisomerization and thermal-isomerization is called “normal photochromism”. When the MC form exists in a highly polar environment, thermal-isomerization from the MC form to the SP form is restricted. In this case, the SP form is only generated upon visible light illumination. Besides, the SP form reverts back to the MC form under dark condition. This is called “reverse photochromism”.

Such fascinating mechanism has been, in the past, deeply investigated after embedding the spiropyran in cationic clays. Spiropyran derivatives in montmorillonite interlayers exhibit both “normal photochromism” and “reverse photochromism” [28,29] depending on the polarity of the interlayers. “Reverse photochromism” is observed when spiropyrans are intercalated into the original (unmodified) montmorillonite. The colored form of spiropyrans requires to be stabilized by the highly polar environment provided with the original montmorillonite. On the other hand, using the organo-montmorillonite (surfactant modified montmorillonite) as a matrix, spiropyrans exhibit “normal photochromism”. In this case, the polarity of montmorillonite interlayers is lowered because of the presence of surfactant molecules bearing generally alkyl chains, which could no longer stabilize the colored form of spiropyrans. These effects are also depending on the preparation methodology (polarity of used solvents and CEC characteristic of the clay) that can affect the delamination extent of unmodified clay, providing normal photochromism behavior. Recently Saso *et al.* [30] have been prepared spiropyran-montmorillonite hybrid materials by Langmuir-Blodgett (LB) approach and intercalation method and the collected results have been compared in terms of optical and morphological features. The hybrid materials show different photochromic behavior in spite of similar composition and chemical environment surrounding the amphiphilic spiropyran; the LB-prepared compounds possess layered structure and exhibit “normal photochromism”; while the intercalated ones, even if with layered structure, show “reverse photochromism” behavior.

By taking into account that no different polarity can be invoked to explain these results, the authors conclude that the SP-montmorillonite hybrid materials are affected by the preparation methodology. In case of the intercalation method, the spontaneous formation of the layered structure takes place in solution. Therefore, SP is intercalated as the MC form that would be stabilized in montmorillonite interlayers. On the other hand, in case of the LB method, the layered structure is formed stepwise by the horizontal dipping technique. Thus, the SP form exists as a major form in montmorillonite interlayers, regardless of the polarity.

**Figure 3:**
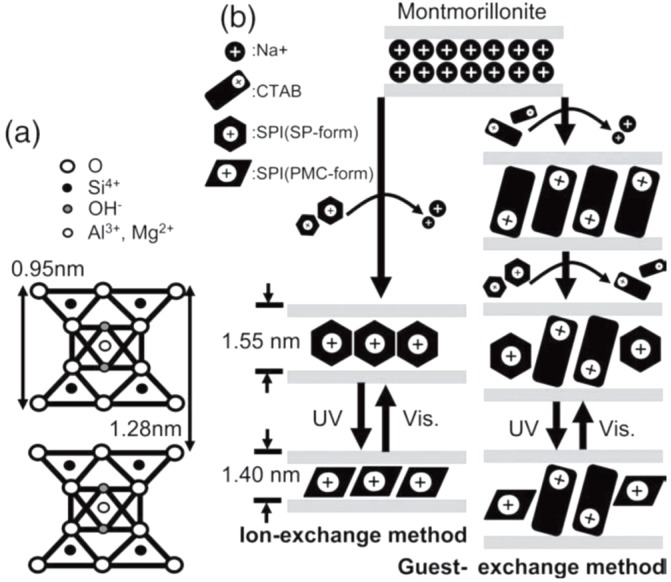
(**a**) The montmorillonite clay structure; (**b**) Mechanism for the intercalation of SPI by the ion- and guest-exchange methods for clay, and the conformational change of SPI in clay interlayers by photoisomerization. This image was published in Thin Solid Films, 518, Kinashi K.; Kita H.; Misaki M.; Koshiba Y.; Ishida K.; Ueda Y.; Ishihara M. Fabrication and optical properties of photochromic compound/clay hybrid films. 651–655, Copyright Elsevier (2009).

“Reversed photochromism” has been observed, instead, for SP-montmorillonite hybrids with both unmodified and modified clay prepared by guest-exchange method [27]. The X-rays (XRD) analysis performed after the exposure of UV irradiation and visible light allows measuring the interlayer distance owing the photochromism mechanism (Figure 3).

When a surfactant (the cetyl trimethylammonium bromide, CTAB) is used as a pre-exchanging reagent, the intercalated CTAB is partially exchanged to SP, and SP in the interlayer undergoes to reversible photoisomerization by UV and visible light. This phenomenon irradiation does not change the basal spacing since the interlayer spacing is already expanded by the coexisted CTAB. When the ion-exchange method is applied to the unmodified clay with high CEC, SP is intercalated directly in the interlayer of silicate sheets and it photoisomerizes reversibly with shrinkage and elongation of the basal spacing creating a sort of a photoresponsive swellable hybrid system.

A similar behavior has been shown by the 2-hydroxychalcones dye (HC), which is converted to the colored flavylium form (FV) under UV irradiation [31]. The immobilization of HC dyes within clay lamellae affects the photochromic response depending on the polarity of the interlayer space as well as the acidity by considering that the FV form prefers the polar environment. The best results in terms of photoinduced coloration are collected by using a previously modified clay with apolar surfactants (to grant the intercalation of the dye not functionalized with exchangeable cation) and an acid as promoter for the conversion of HC to FV species.

Another interesting class of photochromic molecules used as modifiers of a clay are referring to the azobenzene compounds. These dyes show reversible *trans-to-cis* photoisomerization by UV irradiation and subsequent visible light irradiation or thermal treatment. The photoisomerization of azobenzenes in interlayer space of smectites has been extensively studied by Okata *et al.* [32] showing that different microstructures (tilt angle) can be formed, depending upon the layer density of hosts and molecular structure of the dye guest. The intercalated azobenzene chromophore photoisomerizes effectively even in densely packed structures, and the basal spacing changes reversibly upon photoisomerization.

In spite of these interesting results aiming at evidencing the effects of the confinement within cationic clay layers on the photophysical properties of the photochromic dye, the use of collected hybrids in the preparation of thermoplastic polymer-based devices seems to be far from a real application in this field.

**Figure 4:**
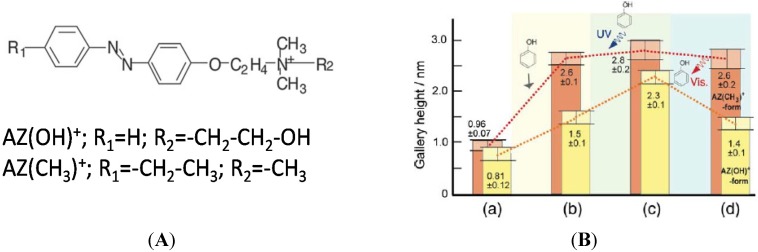
The structure of the AZ-dye used for the clay modification (**A**); The change in the gallery heights of AZ(CH_3_)^+^- and AZ(OH)^+^-montmorillonites (**B**): (a) before the intercalation of phenol, (b) after phenol intercalation, (c) after UV irradiation and (d) after subsequent visible light irradiation This image was published in Applied Clay Science, 40, Okada T.; Sakai H.; Ogawa M. The effect of the molecular structure of a cationic azo dye on the photoinduced intercalation of phenol in a montmorillonite. 187–192, Copyright Elsevier (2008).

Interesting applications involve directly the hybrid systems without the need of their dispersion in polymer matrix. As an example, hybrid materials with intercalated different azobenzene compounds have been exposed to phenol and UV/Vis irradiation [32]. Photochromic behavior together with XRD analysis has been performed to investigate the photoinduced adsorption of the probe molecule (Figure 4).

Both hybrids are able to adsorb and desorb the phenol, but the amount of intercalated phenol depends on the structure/polarity of the dyes. The simple absorption is higher in the case of more apolar AZ(CH_3_), but under UV irradiation the amount of phenol intercalated is higher in the case of AZ(OH) due to larger cis-isomer fraction (more polar with respect to the trans). The relatively smaller polarity of cis-AZ(CH_3_) compared to cis-AZ(OH) may be accounted for the relatively smaller amount of intercalated phenol by the UV. This means that the photoinduced absorption of the phenol is effective only for AZ(OH)-montmorillonte hybrid.

Hybrid materials with NLO properties, have been, instead, prepared [33,34] and successfully dispersed in thermoplastic polymer matrices.

Polymers containing appropriate chromophores show second-order NLO properties when a certain amounts of NLO chromophores are aligned in a noncentrosymmetric manner; indeed large second-order NLO coefficient and excellent time stability are required. In order to meet these needs, a large amount of NLO-active chromophores have to be incorporated into a polymer with a high glass transition temperature (Tg). However, strong dipolar interactions restrict the alignment efficiency of the NLO chromophores in the polymer and consequently result in poor electro-optical (EO) properties. In addition, molecular relaxation of the polymer chains is detrimental to the orientational degree of the NLO chromophores. In order to preserve the NLO properties, the randomization of the poled NLO chromophore has to be prevented and with such purpose the assembly behaviors of organic molecules into layered silicates such as montmorillonite (MMT) could be a convenient route. The dendrons branched backbone (with polyurea(urethane)malonamide core) can be structurally tailored from repeating polymerizations under precise branching control and by incorporating azobenzene dye (dispersed red) at the periphery [33,34,35].

Furthermore, the NLO-active dendrons consisting of secondary amines as reactive points with clay are acidified to form the amine salts, and intercalated into Na+-MMT via ion-exchange to obtain hybrids with different conformation and interlayer spacing whose properties have been studied after dispersion in polyimide samples (PI).

### 2.3. Dispersion of Photoactive Clays in Thermoplastic Polymer Matrices

The dispersion of cationic clays bearing photoactive or photoresponsive molecules is generally performed to simply transfer the photophysical properties of the hybrid to a polymer matrix, to use the optical property as probe in deepening insight some effects (the dispersion level of the clay) or to enhance the photophysical activity by establishing synergic effects with the matrix in building up new photochromic behavior and NLO devices. Only a few examples for each application are following reported.

Clay-rhodamine B, clay-methylene blue, clay-dibenzalidene variously substituted chromophore-hybrids have been dispersed in a polypropylene (PP) matrix to obtain polymer materials with photoresponsive characteristics and with the main purpose to investigate the morphological features through depth analysis of optical responses of chromophore here used as probe/sensor in non-invasive, often real-time, techniques [36,37,38].

**Figure 5:**
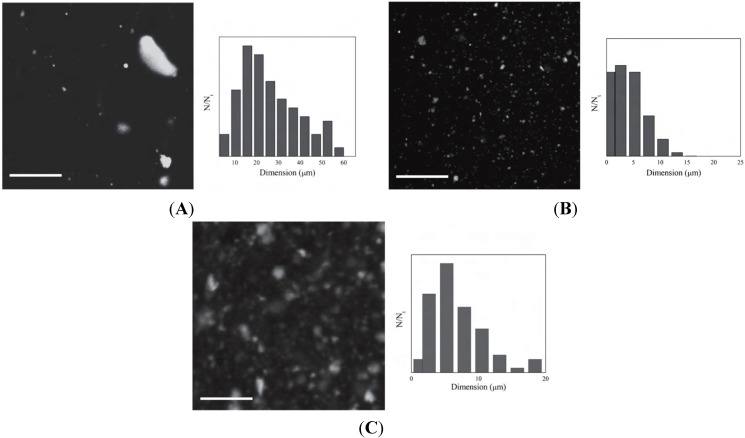
Fluorescence confocal images of polypropylene (PP)/CloNa–R6G (**A**); PP/Clo20A–R6G (**B**) and PP/Clo30B–R6G (**C**). Scale bar corresponds to 100 μm. This image was published in Materials Chemistry and Physics, 123, Aloisi GG.; Elisei F.; Nocchetti M.; Camino G.; Frache A.; Costantino U.; Latterini L. Clay based polymeric composites: Preparation and quality characterization. 372–377, Copyright Elsevier (2010).

Latterini *et al.* [37] prepared three hybrid materials by intercalation of rhodamine (R6G) in clays differing for presence and chemical structure of surfactants: CloNa: unmodified, Clo20A and Clo30B containing respectively methyl, tallow, bis-2-hydroxyethyl ammonium (30 wt%) and dimethyl, dehydrogenated tallow, ammonium (38 wt%). After uptake of the chromophore, followed by an accurate characterization of the structure and of the optical properties, the photoactive hybrids have been melt-dispersed in PP. To reach a direct visualization of the labeled clays into the polymer, the composite materials have been investigated by fluorescence imaging through a confocal microscope, providing pictures with bright particles accounting the dispersion level of the platelets. Size distribution histograms (built up upon measuring the dimensions of the fluorescent particles) have been used to quantify the capacity of solid lamellae to be uniformly dispersed or aggregated into the polymer phase (Figure 5).

The composite materials obtained with CloNa present a very broad size distribution, which appears to be centered around 20 μm (Figure 5a). When labeled Clo20A is used as filler the fluorescent crystals have a much narrow dimension distribution, which is centered at 2.5 μm. The composite obtained by dispersion of the dye-loaded Clo30B compound, shows fluorescent particles with irregular dimensions as proved by the dimension distribution in the 1–20 μm range although centered at 5 μm. This analysis indicates that the dispersion of the inorganic filler in the polymer is closely related to the capacity of the filler to exfoliate, which is likely enhanced for the organically modified Cloisites having enlarged distances between adjacent layers. Even if this result can be ascribed as expected and certainly not new, the preparation and the dispersion in the polymer of labeled inorganic fillers allow to optically visualize the 3-D distribution of all the particles present by making negligible the diffusion of the dye species in the bulk polymer. In fact, the preparation of labeled clays through cation exchange grants the chromophore stay at the interface acting as probe and, therefore, the fluorescence intensity can be analyzed as maps of the filler distribution in the polymeric matrix.

A similar approach has been adopted by Banerjee *et al.* [38] by dispersing a clay labeled with methylene blue in mixtures of PP and a PP sample grafted with maleic anhydride, used here as compatibilizer. In this case the fluorescence spectroscopy analysis of the composites suggests the presence of both dye into the clay and dye dispersed in the polymer matrix. By increasing the mixing time a decrease in intensity of the fluorescence emission peak associated to the dye in the galleries is evidenced. With mixing, more polymer chains are expected to intercalate into the interlayer space (particularly those grafted with maleic anhydride, more compatible with the layered surface) by partially replacing the dye molecules. At the same time a decrease in intensity of the fluorescence emission peak associated to the dye dispersed in the polymer suggests the formation of aggregates owing to interaction between replaced dyes as a result of concentration quenching.

An interesting application of clay-labeling with fluorescent chromophore aiming at investigating the morphological features of nanocomposites, is related to the study of laponite clay nanoparticles diffusion in thermoresponsive poly(N-isopropylacrylamide) (PNIPAM) hydrogels by using wide-field fluorescence spectroscopy (WFS) [39]. These nanocomposite materials are prepared by dispersing the clay as they serve as physical crossinkers to achieve improved material properties. For a deeper insight, the real-time observation of the dynamics of single clay particles on the nano-and microscale, has been performed by fluorescence labeling of the clay nanoparticles with perylenediimide, whose N-hydroxysuccinimide group is reacted with the amino groups on the modified layered surface. The system shows to collapse with temperature, thus allocating the polymer chains much closer to the layered clay surface, by influencing the diffusion coefficient of the clay itself. The result obtained allows, by visualizing the motion of nano-objects in hydrogel, a better understanding of the dynamics within these systems and a better fine-tuning of their ultimate properties.

**Figure 6:**
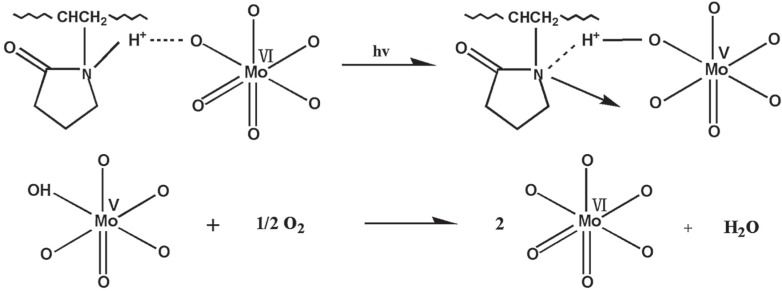
The environment of the photo-reduced site and the process of the photo-reduced reaction. This image was published in Applied Surface Science, 316, Wang X.; Dong Q.; Meng Q.; Yang J-Y.; Feng W.; Han X. Visible-light photochromic nanocomposites thin films based on polyvinylpyrrolidone and polyoxometaltes supported on clay minerals. 637–642, Copyright Elsevier, (2014).

An example of synergic effect between the polymer matrix and the clay in tailoring the photochromic behavior of new nanocomposites has been recently reported by Wang *et al.* [40]. A photochromic phosphomolibdic acid (PMoA) has been entrapped by polar interaction in sodium montmorillonite (Na-MMT) and then the activated clay has been dispersed in polyvinylpirrolidone (PVPd). Normally polymer matrices (PVPd, but also polyacrylamide, polyvinyl alcohol) embedding polyoxomatales can change color in response to light in the UV region. By means of the use of labeled-clay and PVPd the authors claim the preparation of a novel hybrid film with visible-light photochromic properties. The UV-Vis absorption spectra of PMoA/Na-MMT/PVPd film before and after visible-light irradiation (togheter with all the collected results TEM, AFM, TGA, and XPS spectroscopies) were rationalized on the basis of the mechanism described in the Figure 6.

Before visible-light irradiation, any absorption from 400 to 900 nm is observed and the hybrid film is colourless. After visible-light irradiation for 5 min, two broad absorption bands appear, which are attributed to intervalence charge transfer (IVCT) (Mo^5+^→Mo^6+^) at about 810 nm and metal-to-metal *d–d* transition at about 520 nm, respectively. The colour of hybrid films turns from colorless to blue owing to the appearance of heteropolyblues (visible also by AFM). After visible light is turned off, the film starts to bleach gradually in air, but not under N_2_-saturated environment.

The colour still remains the same. If the colored hybrid films are heated up to 80 °C for 30 min, the films turns back their original color. These results show that the hybrid films exhibit excellent bleaching ability with the heating and that the oxygen plays an important role during the bleaching process (reversible photochromism). In particular, the photo oxidation-reduction reactions occur according to a proton charge transfer involving the PVPd and PMoA/Na-Clay and suggesting a synergic effect of the matrix in enhancing the photochromic features.

As already partially discussed in Section 2.2, chromophore containing dendrons intercalated in MMT have been dispersed in polyimide (PI) (based on 2,2-bis(3-amino-4-hydroxyphenyl)hexafluoropropane and 4,4’-oxydiphtalic dianhydride) to obtain hybrid film with NLO properties. The authors exhaustively prove in their numerous papers [33,34,35] that the intercalation in MMT and the successive dispersion in PI provide composite films (on ITO glass) capable of exhibiting optical non linearity without poling, due to the ordered organization of NLO chromophores in the hybrids. This effect is caused by the strong interactions between the closely packed chromophore-containing dendritic structures, and their fixation on the silicate platelets in the same direction. In fact, the reaching of exfoliated morphology is one of the bare need to observe the phenomenon, and the specific polar interactions of clay platelets with the PI plays a key role in enhancing the dispersion level and in stabilizing the reached morphology/chromophore alignment. Accordingly, the NLO properties (with specific reference to electro-optical coefficient, EO) are strongly depending on the d-spacing and dye conformation [35].

The dendrons in layered silicates are capable of undergoing a critical conformational change from tilting (random arrangement) to an ordered structure for specific d-spacing accounting the CEC value of the clay, indicated by the drastic changes of interlayer distances at certain packing densities. In this conformation, the dendrons develop an extended morphology and form a perpendicular conformation (non-centrosymmetric alignment). The electro-optical coefficients (EO) increases sharply from 0 to 6 pm/V while the conformational change occurs and levels off to a limit value suggesting that the degree of ordered morphology remains unchanged for further increase of d-spacing. Furthermore, the addition of a polyimide capable of interaction-induced orientation is found to exert an enhancing effect on the degree of the non-centrosymmetric alignment.

## 3. Layered Double Hydroxides (LDHs)

Among the layered inorganic solids, layered double hydroxides (LDHs), also known as hydrotalcite-like compounds or anionic clays, are host-guest two-dimensional (2D) layered materials consisting of positively charged metal hydroxide layers, acting as host, with hydrated anions intercalated between the layers. The LDH structure can be expressed as [M^II^_1−x_M^III^_x_(OH)_2_](A^n−^)_x/n_·mH_2_O, where M^II^ and M^III^ are divalent and trivalent metals, respectively, and A^n−^ is an n-valent anion [41,42,43].

LDHs can assume a broad range of compositions by varying both the nature and ratio of cations as well as the type of interlayer anions. These last, in particular, can be exchanged by organic anions offering the opportunity to introduce moieties with specific functional properties (e.g., optical and active properties) and increasing the distance between the inorganic layers, which gives rise to an accessible interlayer space on the nanometer scale. Moreover, LDHs have interesting physical and chemical properties due to their structural anisotropy [44,45,46].

The large versatility of LDHs in terms of chemical composition and ability to build up 2D-organized structures has promoted their application in catalysis [47], adsorption [48], medical science [49], polymeric nanocomposites [42,46,50,51] and also as nanostructured materials for photonic and opto-electronic devices [45,52].

In the literature there are numerous examples about the intercalation of organic photofunctional molecules in LDHs (*i.e.*, small molecules, metal complexes, organic ligands or π-conjugated polymers) for applications as dye lasers, solid-state self-emission devices and sensors [52].

The intermolecular interactions inducing non-radiative deactivation processes of fluorescent dyes in the solid state (*i.e.*, fluorescence quenching) can be reduced by confinement of the dyes between the LDH layers. This method offers a series of synergistic effects and advantages, such as the enhancement of thermal, optical, and physicochemical properties of the guest molecules. The LDH matrix provides chromophore molecules with a confined and stable environment, which reduces molecular thermal agitation (intermolecular collisions, vibrations, and rotations, *etc.*) and improves fluorescence efficiency; the chromophore aggregation in the LDH matrix is then inhibited by host-guest interactions (*i.e.*, electrostatic attraction, hydrogen bonding), and the fluorescence quenching is reduced, as well summarized in previous papers and reviews [43,44,45,46,52,53,54].

Therefore, our attention is here focused on the main advances achieved in the last five years about the preparation of these hybrid systems and their sensor applications, considering also polymer based functional LDH nanocomposites.

### 3.1. LDHs Functionalization by Intercalation: Advances in the Preparation Methods

The intercalation of organic anions between LDH layers is generally achieved by anion exchange or co-precipitation. With both the methods the dyes can be successfully intercalated as evidenced in the case of colorants to produce colour and multicolor organic-inorganic hybrid pigments [55,56,57,58,59,60], azo-dyes [61], fluorescein [62,63,64], pyrene and perylene derivatives [65,66], stilbene and anthracene derivatives [67,68,69]. However, as already mentioned, the optimization of the optical properties requires dyes molecular dispersion within the galleries. Accordingly, in the last years, one of the main challenges has been that to find effective solutions to overcome this problem. In the following, there are reported a few of the most recent examples of photofunctional-LDHs prepared by conventional approaches as well as by innovative synthesis showing advanced optical properties with respect to the neat dye.

One of the most successful methods for achieving the disaggregation of the dyes is that of the co-intercalation with a second organic anion, which is generally a surfactant with a long aliphatic tail [70,71,72,73,74]. In this way the distance between the dyes is increased, thus avoiding the aggregation; the environment is homogeneous and non-polar, thus enhancing the luminescence; the surfactant can be pre-intercalated to enlarge the interlayer spacing, thus facilitating the successive intercalation of the bulky dye anions. Finally, the needed amount of chromophore is reduced with considerable cost-saving.

There are numerous examples of application of this methodology. One of the most clarifying involves the fluorescein (FLU) sodium salt. This last can be directly loaded into LDH layers by anion exchange, but in this way the FLU anions uncontrollably fill in the gallery or attach at the surface of the LDH nanoparticles with very high local concentration. This leads to close spacing between molecules and the resultant hybrid materials retain very low or even no fluorescence. In contrast, excellent results were achieved by co-intercalating FLU anions and alkyl sulfonates with different alkyl chain lengths (C_n_H_2n+1_SO_3_, with n = 5, 6, 7, 10, and 12) into the LDH galleries [70]. It was found that the surfactant molecules reduce the fluorescence quenching by inhibiting non-radiative processes and influencing the orientation order and aggregation characteristics of the dye molecules.

**Figure 7:**
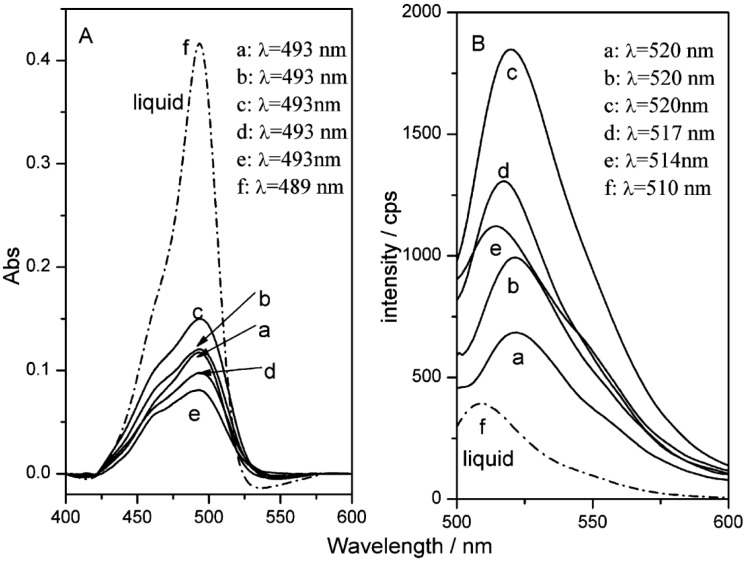
(**A**) UV-Vis absorption spectra and (**B**) photoemission spectra (excitation wavelength of 4890 nm) of FLU-C_n_H_2n+1_SO_3_/layered double hydroxide (LDH) thin film with (a)–(e) (n = 5, 6, 7, 10, 12) and (f) pristine fluorescein (FLU) solution. Reprinted with permission from Shi *et al.* [70]. Copyright American Chemical Society (2010).

Interestingly thin films of the co-intercalated FLU-C_n_H_2n+1_ SO_3_/LDHs hybrids were prepared by solvent evaporation on Si substrates. Their analysis evidenced that the orientation of FLU, as well as the anisotropy, the fluorescence wavelength, the fluorescence quantum yield, and the lifetime correlate all with the microenvironment of the LDH gallery. Notably all those features can be tuned by simply changing the alkyl chain length of the surfactant. Optimal parameters were obtained with n = 7 as number of carbons of the surfactant chain, due to the “size-matching” rule between the organic dye and surfactant (Figure 7) [70,72,73,74].

More recently a new method of preparation of photoactive LDH-FLU thin films based on the self-assembly of the LDH nanocrystals and on well-controlled intercalation of the dye has been proposed [62]. First an oriented film made of LDH in carbonate form (LDH-C) was deposited on a Si substrate by ultrasonic treatment of the substrate in 1-butanol containing the LDH-C powder. Then the LDH film with the intercalated FLU (LDH-FLU) was obtained by treatment of the LDH-C film with FLU dyes in an ethanol–toluene mixture at 120 °C for 48 h.

The experimental conditions adopted in this work allowed the carbonates to be successfully replaced with the FLU molecules in the gallery spaces of the LDH-C. The intercalation of FLU dyes was, indeed, induced by the ethanol, which provided solubility for the de-protonation of FLU to the anionic form and for the fast diffusion of the FLU into the LDH interlayers. X-rays diffraction (XRD) evidenced the highly oriented interlayer arrangement of the dianionic form of the FLU in the LDH interlayers. The dye molecules were electrostatically immobilized between the positively charged LDH layers with a monolayer packing structure, as confirmed by theoretical calculations and absorption spectrum of the LDH-FLU film.

Yan *et al.* [75] proposed another novel approach to overcome the problem of the dye aggregation. The method is based on the covalent bonding of FLU anions into the LDH nanoparticles (LDH-Co-FLU) so that certain spacing between fluorophores can be maintained. The results evidenced that this hybrid has much higher fluorescence quantum efficiency than those prepared using anion exchange and co-precipitation approaches (55.1% with respect to 3% and 12.4% respectively. Moreover, some important characteristics for the application of LDH-Co-FLU in optical devices were found: the fluorescence intensity was proportional to the concentration in a certain range, which is an excellent characteristic for quantitative applications; the nanohybrid remained fluorescent even in a dry powder form, and it could self-assemble into a transparent and free-standing film, fluorescent under UV light. Similarly, an organic oligothiophene fluorescent compound (N-(3-(triethoxysilyl)propyl)-[2,2’:5’,2’’:5’’,2’’’-quaterthiophene]-5-carboxamide, T_4_Si) was covalently attached to a ZnAl-LDH previously modified by using direct microwave (MW)-assisted silylation [76]. In this case, the fluorescent dye was not intercalated between the layers, but grafted on the outside of the nanoparticles. This procedure enables surface modification while preserves LDH interlayer region, and the grafted dye quantity can be regulated by the MW irradiation time. Filmability, fluorescent properties, and biocompatibility of the silylated compound were demonstrated thus highlighting the potential of the so obtained lamellar nanoparticles in applications ranging from diagnostic biomedical tools to photonics and sensing.

The possibility to delaminate LDH microcrystals into nanosheets is another effective and fascinating method to fabricate ordered nanostructured thin film by electrostatic layer-by layer (LBL) assembly [77] There are several intriguing examples about the use of this method. The first is that proposed by Han *et al.* [78] that prepared cobalt phthalocyanine/layered LDH ultrathin films with a long-range ordered structure and uniform deposition. Ma *et al.* assembled an optical brightener, such as the anionic stilbene derivative tetrasodium 4,4’-bis[2-di(b-hydroxyethyl)amino-4-(4-sulfophenylamino)-s-triazin-6-ylamino] stilbene-2,2’-disulfonate (BBU), by the LBL with MgAl-LDH nanosheets [79]. The UV-Vis absorption and fluorescence spectroscopy showed an orderly growth of the BBU/LDH films upon increasing the number of deposition cycles.

There are also examples of successful intercalation of polyanions by LBL, like in the case of the preparation of polyaniline/LDH [80] and poly(p-phenylene)/LDH ultrathin films [81]. In this last case the sulfonated π-conjugated polymer (π-CP) was alternatively deposited on LDH layers to obtain ultrathin films with a well-defined blue fluorescence and long-range order.

**Figure 8:**
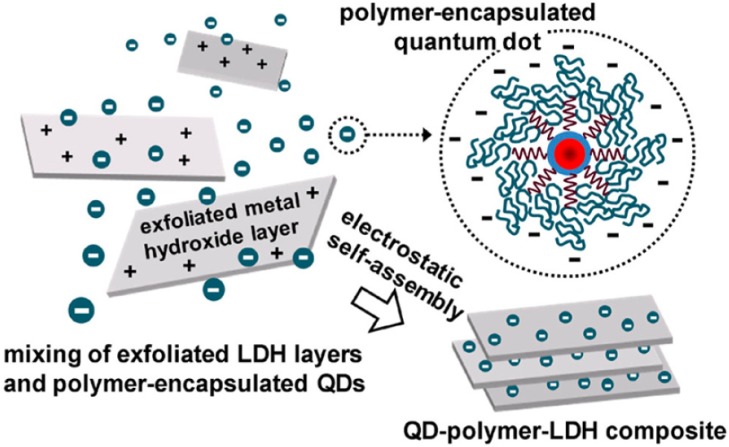
Schematic illustration of the assembly process for the formation of quantum dot (QD)-polymer-LDH composites. Reprinted with permission from Cho *et al.* [82]. American Chemical Society (2013).

The method of the LBL assembly was also proposed as a strategy for the incorporation of quantum dots (QDs) into LDH without deterioration of the photoluminescence efficiency of the QDs thus attaining highly luminescent and photostable composites [82]. The QDs were synthesized in an organic solvent and then encapsulated by poly(maleic acid-alt-octadecene). The polymer-encapsulated QDs with negative zeta potentials were electrostatically assembled with positively charged LDH nanosheets to form QD-polymer-LDH composites (Figure 8).

**Figure 9:**
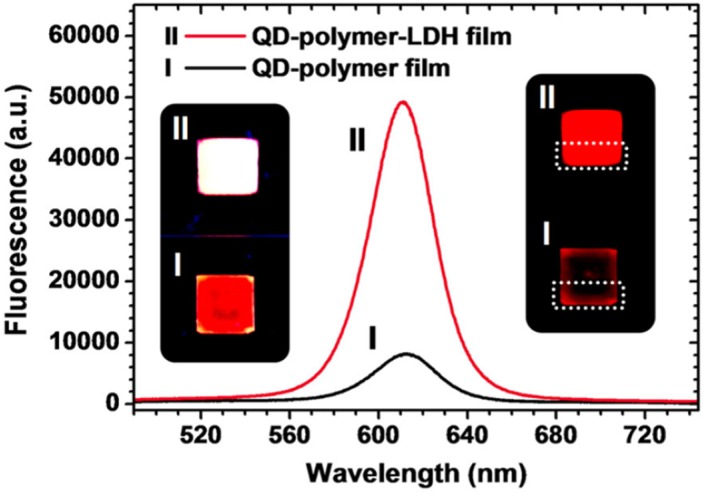
Photoluminescence spectra of films of (I) QD-polymer solution and (II) QD-polymer-LDH composite solution. (left inset) Photograph of the QD-polymer film and the (right inset) QD-polymer LDH composite film under 60 microW/cm^2^ power UV light irradiation. Photograph of the films under lower power UV light irradiation for colour recognition. Reprinted with permission from Cho *et al.* [82]. Copyright American Chemical Society (2013).

It was found that the photoluminescence properties of the hybrid films preserve those of the organic QD solutions and that the QD-polymer-LDH composites affords enhanced photostability through multiple protections of the QD surface by polymers and LDH nanosheets from the environment. Interestingly, the fluorescent spectrum of the composite did not change compared to the colloidal form while the QDs and the polymer-encapsulated QDs without LDH composite formations were red-shifted by isolation from the colloidal state. In contrast, it was observed a photoluminescence quantum yield reduction of QD-polymer film (Figure 9), which is due to a non-uniform distribution of the fluorophores. These QD-polymer-LDH composites have a variety of potential application areas such as lighting, display, and optical coating materials.

The possibility to prepare ultrathin films of photofunctional LDHs makes these systems particularly attractive for application as pH-sensors, electrochemical sensors, sensors for volatile organic compounds (VOCs) and biological molecules, as well as sensors for the identification of hazardous molecules.

Shi *et al.* [83] demonstrated the possible use of the co-intercalated FLU-C_n_H_2n+1_SO_3_/LDH (with n = 7) [70,72] as an optical pH sensor. A highly oriented photoluminescent film (polarized with anisotropy value r = 0.29) obtained by electrophoretic deposition was tested showing a broad linear dynamic range for solution pH (5.02–8.54), good repeatability (relative standard deviation, RSD, less than 1.5% in 20 consecutive cycles) and reversibility (RSD less than 1.5% in 20 cycles), as well as photostability and storage stability (ca. 95.2% of its initial fluorescence intensity remains after one month) as well as fast response time (2 s). The pH sensor was measured in solution with pH 5.02, 6.51, and 8.54. No obvious signal drift was found for the fluorescence intensity at the maximum emission peak and good repeatability over 20 consecutive cycles was obtained. Moreover, the reversibility of the pH sensor was demonstrated by alternate immersion into two solutions with pH 5.02 and 8.54. Finally, no trace of leached FLU was detected in the measured solution that is an interesting result considering that the leaching of the dye over prolonged periods generally leads to unreproducible measurements.

A significant electrocatalytic performance for the oxidation of dopamine was also found for cobalt phthalocyanine/layered LDH ultrathin films [78] and for the co-intercalated FLU-C_n_H_2n+1_SO_3_/LDH (n = 7), which was used as electrode surface modifier for preparing electrodes with rather high sensitivity and selectivity [72]. Interestingly, in this last case it was demonstrated a correlation between the hybrid sample exhibiting the strongest luminous intensity and that showing the best electrochemical behaviour for dopamine, thus indicating that the electrogenerated chemiluminescence of the FLU accords with the photoluminescence of FLU itself.

**Figure 10:**
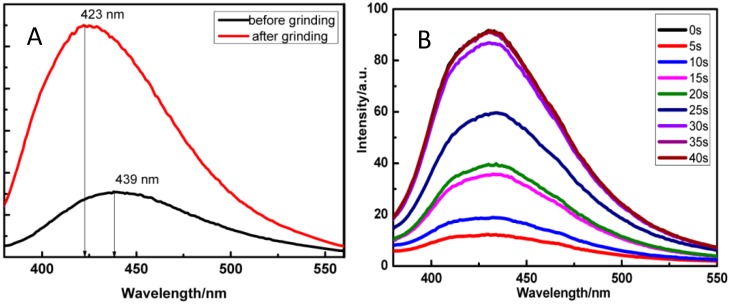
(**A**) Fluorescence spectra of the niflumic acid (NFC)-heptanesulfonate (HPS)/LDH sample containing 5% NFC before and after grinding; (**B**) In situ time-dependent monitoring of the fluorescence of the NFC-HPS/LDH sample containing 5% NFC responding to THF. Reprinted with permission from Zhao *et al.* [84]. Copyright American Chemical Society (2014).

An interesting hybrid LDH with sensing application is the material proposed by Zhao *et al.* [84] which reported the assembly of a typical aggregation-induced-emissive (AIE) molecule, such as the niflumic acid (NFC), into the interlayer region of a ZnAl-LDH with heptanesulfonate (HPS) as a co-intercalating guest. This system showed a mechano-induced and solvent stimuli-responsive luminescent change. The NFC is, indeed, a flexible molecule containing a rotatable aromatic amine unit, which presents AIE property with the photoluminescence quantum yield values of 0.03% and 4.26% for solution and solid state respectively. However, pure NFC cannot exhibit mechano-induced fluorescent change due to the highly ordered H-bonding network within the molecular solid, for which it is difficult to modify the intermolecular interactions. In contrast, the fluorescence spectra of the NFC-HPS/LDH composites evidence that the sample with 5% NFC with respect to the interlayer guests had an optimal luminescent intensity and shows the most luminescent mechano-response after grinding (Figure 10). Moreover, the NFC-HPS/LDH (5%) also presents reversible luminescent response to different VOCs (such as tetrahydrofuran, methanol, acetone, toluene, and chloroform).

**Figure 11:**
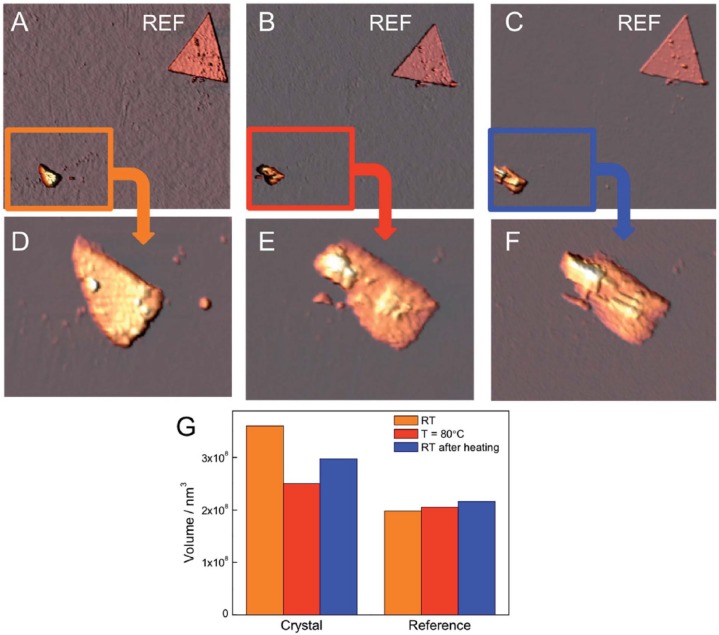
AFM images of the CoAl–LDH-AO5 acquired at room temperature (RT) (**A**); at 80 °C (**B**); and again at RT after the heating (**C**); In each image it is possible to distinguish a crystal and a reference marker. Image size 17 micron × 17 micron. (**D**), (**E**), and (**F**) correpond to magnifications of (**A**), (**B**), and (**C**) respectively to observe in detail the evolution of the shape of the crystal. (**G**) Histogram of the volume of CoAl–LDH-AO5 hybrid system and of the reference measured by AFM. Reprinted with permission from Abellan *et al.* [85]. Copyright Royal Society of Chemistry (2015).

Very recently it has been also proposed the co-intercalation of the thermoresponsive 4-(4-anilinophenylazo) benzenesulfonate (AO5) with sodium dodecylsulfate (SDS) surfactant into a ferromagnetic CoAl-LDH. This process provides a hybrid material exhibiting thermochromism due to the isomerization between the azo (prevalent at room temperature) and the hydrazone (favored at higher temperatures) tautomers [85]. These hybrids show thermally induced motion triggering remarkable changes in both crystal morphology and volume thus behaving like “thermoresponsive breathing materials”. The volume change vs. temperature was demonstrated at the nanoscale level by XRD analysis carried out on thin films at different temperatures. It was observed that the reversible change into the two tautomers of AO5 is reflected in a shift of the position of the diffraction peaks at high temperatures towards lower interlayer spacing for the hydrazone form. In addition, it produces a broadening of the peaks reflecting lower crystallinity and ordering due to non-uniform spacing between the layers. At the microscale level, it was investigated the variation in the morphology of the CoAl–LDH-AO5 crystals (as thin films deposited on a silicon wafer) by means of a variable temperature AFM. An evolution of the shape of the crystals moving from room temperature to 80 °C and then cooling again was reported (Figure 11). Upon heating the particle showed a pronounced compression, which was partially reverted upon freezing to room temperature. Thus, a large quasi-reversible change in the volume up to about 25% was observed, which is correlated to a sliding movement of the hydroxide sheets.

In addition, it was demonstrated that the magnetic response of the hybrid can be modulated due to the thermotropism of the organic component that tunes the magnetism of the CoAl–LDH sheets in a certain range by influencing the distance and in-plane correlation of the inorganic LDH. However, the magnetic properties were much less affected than in the case in which the functional molecules are bridging adjacent layers like in the case of the photoisomerizable trans-azobenzene-4,4’-dicarboxylic acid [86].

Another interesting sensor application of hybrid LDHs is the identification of nitroaromatic explosives which was observed for LDH ultrathin films containing the BBU optical brightener [41]. For this composite it was found a well-defined one/two-photon polarized photoemission and a fast, selective and reversible luminescence response to nitroaromatic molecules, with the most significant luminescent red-shift and quenching occurring for picric acid.

Finally, it is noteworthy that thin films of a hybrid fluorescent LDH obtained via co-intercalation of 2-phenylbenzimidazole-5-sulfonate (PBS) (a UV light absorber) and 1-decane sulfonate (DES) anions [87] showed a remarkable transformation (violet to UV light) for nucleotide triphosphates compared with their diphosphate and monophosphate counterparts, which makes this material a potential sensor for nucleotide molecules.

### 3.2. Polymer/LDH Nanocomposites: Sensing Properties

At present, polymer/LDH nanocomposites showing sensing properties have been prepared by intercalation of polyanions or LBL self-assembly technique, thus obtaining samples at a small level.

An example of LDH polymer based nanocomposite used for preparing a non-enzymatic sensor for the determination of hydrogen peroxide is that proposed by Jin *et al.* [88]. The system is based on the intercalation of Prussian blue (Fe_4_^III^(Fe^II^(CN)_6_, PB) in LDH. In particular nanostructured PB exhibits excellent electro-reduction of H_2_O_2_ due to its electro-catalytic activity, low detection limit and good selectivity. PB nanoparticles assembled between LDH layers were obtained by intercalation of Fe(CN)_6_^4−^ ions via anion exchange followed by Fe^3+^ addition. The hybrid (LDH-PB) showed an enhanced electrochemical response because the electrochemical active sites were increased, but if deposited on a glassy carbon electrode (GCE) in this form it shrunk and cracked. Accordingly LDH-PB/poly(styrene sulfonate) (PSS) composites were prepared conglutinated on the GCE by polyaniline (PANI) to achieve a novel hydrogen peroxide sensor with high sensitivity, low cost and good stability. First a film of PANI was deposited on the electrode, later a solution containing LDH-PB/PSS was deposited on the PANI film thus obtaining the LDH-PB/PSS/PANI/GCE system. The fabricated electrode showed a well-defined pair of redox peaks and excellent electrocatalytic activity. The sensor response to H_2_O_2_ showed a linear range of 6 × 10^−6^–1.86 × 10^−4^ M with a low detection limit (0.38 μM).

**Figure 12:**
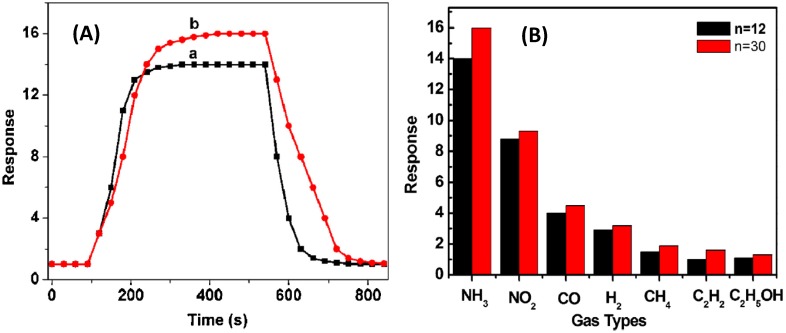
(**A**) Response and recovery curve of PANI/LDH multilayer films (n = 12 and 30, respectively) to 1000 ppm ammonia at RT; (**B**) Gas response of PANI/LDH multilayer films to 1000 ppm ammonia and 10,000 ppm of NO_2_, H_2_, CO, CH_4_, C_2_H_2_ and ethanol at RT. Reprinted with permission from Xu *et al.* [80]. These images were published in Journal of Hazardous Materials, 262, Xu D.-M.; Guan M.-Y.; Xu Q.-H.; Guo Y. Multilayer films of layered double hydroxide/polyaniline and their ammonia sensing behavior. 64–70, Copyright Elsevier (2013).

Similarly, the polyaniline (PANI)/LDH multilayer system [80] obtained by LBL approach was tested as gas sensor for ammonia. In comparison with pure PANI, which cannot be used as gas sensor due to its poor processing performance, the PANI/LDH intercalated system shows an improved ammonia response because the LDH layers provide a confined and stable environment for the immobilization of PANI. Also it increased the reaction spaces between PANI and gas molecules. The ammonia-sensing behaviour of the multilayer films was observed by measuring the resistance change when the multilayer films were exposed to NH_3_. In Figure 12 it can be observed the response and recovery curves of two different multilayer systems made of 12 and 30 layers, respectively. After the film exposure to NH_3_ the response value increased with the layers number. Moreover these multilayer films can detect ammonia gas down to 100 ppm even if the response towards other gases is relatively low.

Another example of polymer/LDH nanocomposite system useful as luminescence probe or sensor in chemical and biological systems is that proposed by Bach *et al.* [89] and based on the doping of a poly(ε-caprolactone) (PCL) grafted LDH system with terbium ions (Tb^3+^). By combining the ring opening polymerization, click chemistry, and coordination chemistry a complex system was prepared with the PCL covalently grafted to the LDH and Tb^3+^ coordinated to this system in the presence of 1,10-phenanthroline (Phen) (LDH-g-PCL-Tb^3+^- Phen).

The LDH-g-PCL-Tb^3+^-Phen hybrid showed four emission bands with high fluorescence intensity with excitation at 328 nm. It was found that the emission intensity of LDH-g-PCL-Tb^3+^-Phen complexes at 546 nm is 2 times higher than that of Tb^3+^-Phen thus concluding that the LDH-g-PCL works like a macromolecular ligand coordinating with Tb^3+^ ions and it also creates a stable rigid structure. This feature is advantageous for the inhibition of non-radiative transition and enhance the fluorescence emission intensity.

**Figure 13:**
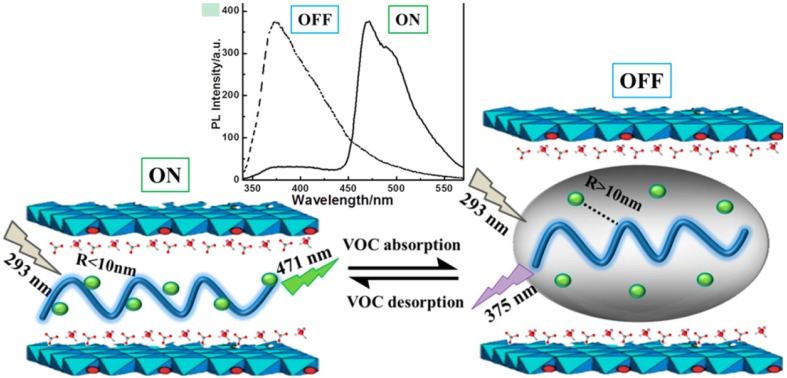
LBL assembling of the poly(N-vinylcarbazole) (PVK) and Ir(F_2_ppy)_3_ between LDH nanosheets. Föster resonance energy transfer (FRET) process between PVK and Ir(F_2_ppy)_3_ interrupted by VOC vapors. Fluorescence spectra in the atmosphere (black) and in toluene vapor (red) excited at 294 nm. Reprinted with permission from Qin *et al.* [90]. Copyright American Chemical Society (2014).

In the field of chemical sensors for harmful organic solvents, it is particularly interesting the system developed by Qin *et al.* [90] and based on the LBL assembling of the fluorescent poly(N-vinylcarbazole) (PVK) and phosphorescent tris[2-(4,6,difluorophenyl)pyridinato-C^2^,N] iridium (III) (Ir(F_2_ppy)_3_) between LDH nanosheets to form ordered ultrathin films. In this 2D system it is established a Föster resonance energy transfer (FRET) process between PVK (donor D) and Ir(F_2_ppy)_3_ (acceptor A), which is based on the transfer of the excited-state energy from a donor to a proximal acceptor, and strongly depends from the donor-acceptor distance. Interestingly, the system works as sensor because the FRET process is interrupted, when the hybrid system comes in contact with common VOCs. Therefore, the system shows an ON/OFF fluorescence signal when contacting VOC vapours because the VOC vapours penetrate the interlayers increasing the space between the D/A pair and accordingly the FRET process is interrupted; however, when the film is back into dry air the FRET behavior is recovered (Figure 13).

The sulfonated poly(p-phenylene) anion/LDH system obtained by the LBL electrostatic assembly approach by Yan *et al.* is an example or polymer-LDH nanocomposite material [81]. The collected data evidenced that the LDH layers improve the luminescence properties of the π-CP by avoiding the formation of π–π stacking interactions and the LDH monolayers led to higher UV photostability for its blue luminescence. The authors indicated that by varying the alignment and component of polymer and LDH monolayers the system can be tuned and controlled to make the ultrathin films much flexible and potential for the design of various optoelectrical devices.

## 4. Noble Metal Nanoparticles (NMNP) Thermoplastic Polymer Sensors

Polymer films, suitable for sensing application, can be produced by dispersing noble metal nanoparticles (NMNP) into thermoplastic polymers. The combination of their optical properties with the mechanical ones of thermoplastic host materials has recently received a lot of interest [91,92,93,94,95].

Wide varieties of physical and chemical procedures have been developed in order to synthesize nanoparticles of different compositions, sizes, shapes and controlled polydispersity, such as chemical reduction [96,97], photochemical reduction [98], laser ablation [99], electrochemistry [100], microwave irradiation [101], lithography [102] or high energy irradiation [103]. In addition, with the growing need to minimize or eliminate the use of environmental-risk substances, as the green chemistry principles describe, the synthesis of nanoparticles using biological entities has received increasing attention in the last decade [104].

The optical properties, of clusters of noble metals, such as gold, silver or copper, differently from smooth metal surfaces or metal powders assume a real and natural color due to the absorption of visible light at the surface plasmon resonance frequency, and this, as described by the Drude-Lorentz-Sommerfeld theory is much affected by cluster size [105,106,107].

Noble metal nanoparticles incorporated in polymeric matrices may confer to the derived thin films tuneable absorption and scattering characteristics, which depend on particle size, shape and aggregation [108]. In particular, the decrease in metal particle size leads to broadening of the absorption band, decrease of the maximum intensity and often to a hypsochromic (blue) shift of the peak, and these effects depend also on cluster topology and packing.

When dispersed into polymers as non-aggregated form, nanoparticles with very small diameters (a few nm) allow the design of materials with much reduced light scattering properties, overcoming the widely encountered problem of opacity of heterogeneous composites for optical applications. Even more interesting is the fact that nanoparticle dispersions in a polymer matrix can be rendered macroscopically anisotropic, a feature that has allowed their use in nonlinear optical devices and linear absorbing polarizers, e.g., for display applications [109,110].

The unique physicochemical properties of such metals at the nanoscale have led to the development of a wide variety of biosensors, such as: (i) nanobiosensors for point of care disease diagnosis; (ii) nanoprobes for in vivo sensing/imaging, cell tracking and monitoring disease pathogenesis or therapy monitoring and (iii) other nanotechnology-based tools that benefit scientific research on basic biology.

These applications are reported by Doria *et al.* in a recent comprehensive review [111] and will not be described in more detail in this review for space reasons.

### 4.1. NMNP/Polymer Composites: Preparation Methods

Several methods have been reported for the preparation of NMNP polymer composites [91,112,113].

The most common procedure to obtain a dispersion of MNPs in a polymer matrix is to prepare a colloidal solution of stabilized MNPs and then to mix it with the desired polymer in a mutual solvent and cast a film by evaporation from the solution [114]. In contrast, few examples are reported showing the dispersion of preformed MNPs in a polymer matrix by melt mixing at high temperature [115,116]. Usually a water-soluble metal salt is dispersed into an organic solvent using a tetraalkylammonium bromide as phase transfer agent and successively reduced with sodium borohydride in the presence of an alkylthiol as surface stabilizer to prevent coalescence of growing nanoparticles [97,117]. In addition to thiols, different surface stabilizers have been used such as amines, poly(vinyl pyrrolidone) (PVP) and poly(sodium acrylate) [118,119,120]. By using the colloid chemistry technique described above, MNPs have been dispersed in UHMWPE [121,122], HDPE [123], PVA [124,125], polydimethylsiloxane [126] and poly(styrene-*block*-ethylene/ propylene) [127].

Another approach for the preparation of nanocomposite films containing metal nanoparticles involves the in situ formation of the nanoparticles directly within the polymer matrix [97,128]. This process is simple and just requires the reduction of the metal ions precursors by a photochemical or a thermal-induced process. Recently, polymeric films based on poly(vinyl alcohol) and poly(ethylene)-co-(vinyl alcohol) matrices and nanostructured gold have been prepared by an UV photo-reduction process [129].

**Figure 14:**
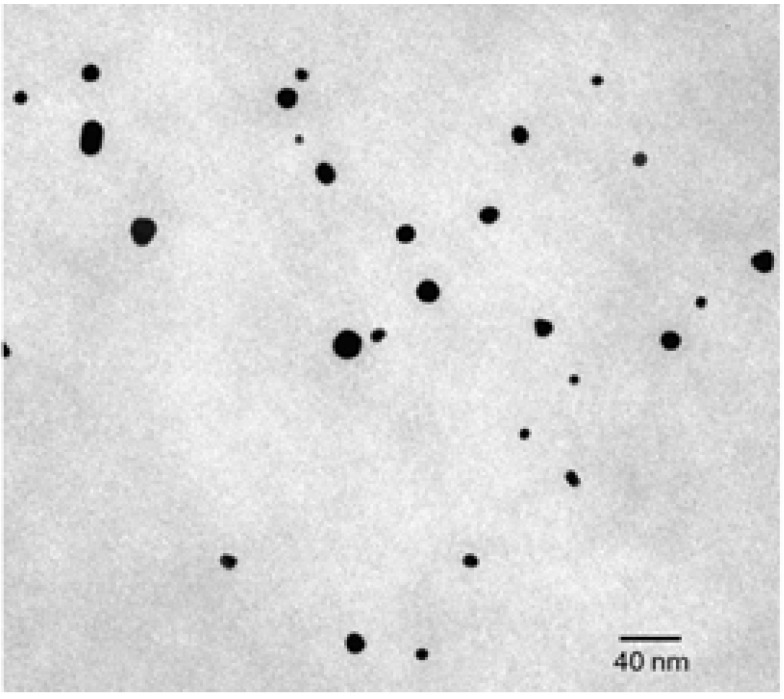
Bright-field transmission electron micrograph of Au/PVAl film irradiated for 5 min. Reproduced from Pucci *et al.* [129] by permission of The Royal Society of Chemistry.

In this case the polymer matrix, based on vinyl alcohol repeating units, acts as co-reducing agent, as protective agent against particle agglomeration and as macroscopic support. The very fast process provided gold nanoparticles with average diameters ranging from 3 to 20 nm depending on the host polymer matrix and the irradiation time (Figure 14).

The preparation of silver “nano-dispersion” directly in the PVA matrix by a one-step method based on the reduction of the inorganic precursor through a solid state synthesis has been achieved by thermal annealing [130] and UV irradiation [131,132,133], which result very efficient methodologies because they take advantage on the formation of a complex between the PVA matrix and the silver nitrate: Ag^+^ ions can be easily chelated by the hydroxyl groups of the polymer and then reduced directly in the host matrix.

The synthesis of polymer/AgNP hybrid nanocoposites under microwave irradiation [101], in supercritical carbon dioxide medium [134] and by miniemulsion encapsulation method [135] is also reported.

### 4.2. NMNP/Polymer Composites: Sensing Properties

#### 4.2.1. Chemiresistor Sensors

In the literature one can find several studies on the preparation of NMNP/polymer composites based on thermoplastic and/or conductive polymer matrices, whose electrical resistance characteristics are the basis for their application as sensors for the detection of organic substances, also biological.

A nanocomposite with a core-shell structure containing polystyrene (PS), PANI, and Au nanoparticles (AuNPs) was synthesized by Liu *et al.* [136] and characterized by microscopic and spectroscopic investigations. The cyclic voltammetric results of a nanocomposite-modified glassy carbon electrode (GCE) indicated that this material was highly electroactive thanks to a decrease in the percolation threshold between its components. The electrode showed redox activity in a wide pH range from 1.0 to 9.0. Due to the excellent electrochemical behavior and the good biocompatibility, the resultant nanocomposite is quite suitable for the construction of biosensors. As a model, glucose oxidase (GOD) was entrapped onto the nanocomposite-modified electrode. The direct electron transfer between GOD and the electrode has been easily realized and the enzyme exhibited bioactivity in solutions with a widerange of pH and, therefore, promising as sensor for glucose detection.

In a recent paper [137], a highly stable nanocomposite film based on embedding gold nanoparticles (nanoAus) into a poly(3,4-ethylenedioxythiophene) (PEDOT) modified Pt electrode was fabricated and explored for dopamine sensing. The PEDOT film was synthesized in 1-butyl-3-methylimidazolium tetrafluoroborate as ionic liquid. It was found that PEDOT film exhibited a fibrillar network-like structure with the pore size from 50 to 100 nm. This network-like structure provided an open ion accessible structure, which was convenient to entrap the foreign material and yield a composite. Citrate coated AuNPs with average diameter of 16 nm have been immobilized on the polymer matrix via electrostatic interactions as shown by SEM images of nanoAus/PEDOT composite. This resulting morphology facilitates the mass transport and weakens the capacitive current. This modified electrode took advantage of the high stability and excellent permselectivity of PEDOT, and exhibited a wide linear response to dopamine from 6.0 × 10^−6^ to 0.013 M. The detection limit was 0.2 μM (*s*/*n* = 3) and the amperometric response time was 2.5 s. Over the 8 months period of this study, the nanocomposite-modified electrode still retained 85% of the original current response to dopamine. The highly stable modified electrode with improved sensitivity and selectivity could provide an ideal matrix for commercial applications.

Barahona *et al.* reported a new format of apta-sensing hybrid composite particles for SERS detection of malathion using Surface-Enhanced Raman Spectroscopy (SERS) [138]. The authors developed new polymer-AuNP-aptamer microspheres that combine extraction capability by aptamer-target analyte interaction and Raman signal enhancer for SERS detection of the malathion pesticide. Working under described experimental conditions, the polymer-AuNP aptamer successfully allows the direct detection of malathion at 3.3 lg mL^−1^ The apta-sensing microspheres are a system wellsuited for industrial and agricultural applications, as only basic equipment is required for analyte separation.

**Figure 15:**
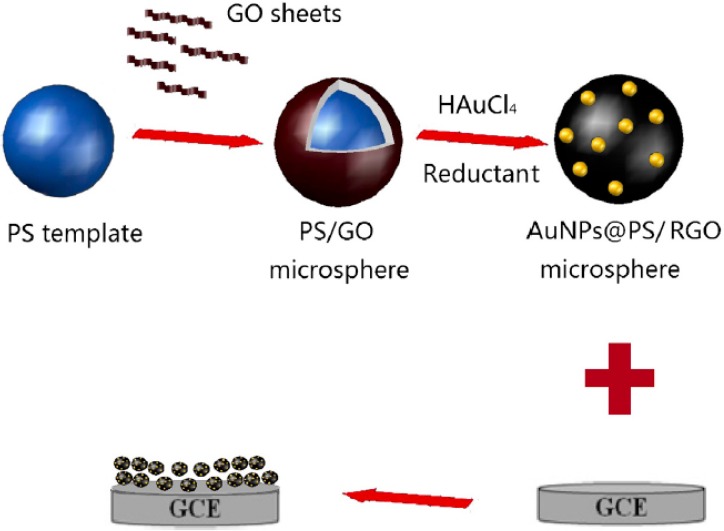
Scheme showing the chemical route to the synthesis of AuNPs@PS/RGO. This image was published in Colloids and surfaces. B, Biointerfaces, 112, Qian T.; Yu C.; Wu S.; Shen J. Gold nanoparticles coated polystyrene/reduced graphite oxide microspheres with improved dispersibility and electrical conductivity for dopamine detection. 310–314, Copyright Elsevier (2013).

A core-shell polystyrene/reduced graphite oxide composite decorated with AuNP (AuNPs@PS/RGO) (Figure 15) has been successfully prepared by Qjan *et al.* [139]. The decorative AuNPs could prevent the aggregation of RGO by electrostatic repulsive interaction, thus leading to a highly homogeneous composite.

**Figure 16:**
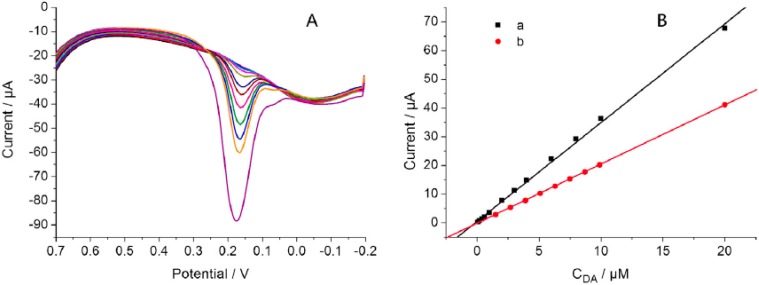
(**A**) The DPVs of increasing DA concentration in 0.1 MPBS (pH = 6.5), DA concentration was 0.05, 0.1, 0.2, 0.4, 0.6, 1, 2, 3, 4, 6, 8, 10, and 20 μM (from top to bottom), respectively; (**B**) The calibration curve of DA obtained with (a) AuNPs@PS/RGO, and (b) PS/RGO modified GCE. This image was published in Colloids and surfaces. B, Biointerfaces, 112, Qian T.; Yu C.; Wu S.; Shen J. Gold nanoparticles coated polystyrene/reduced graphite oxide microspheres with improved dispersibility and electrical conductivity for dopamine detection. 310–314, Copyright Elsevier (2013).

The electrochemical test results show that the AuNPs@PS/RGO composite modified electrode exhibits excellent sensitivity and selectivity response for dopamine (DA) (Figure 16). Moreover, this electrochemical biosensor is suitable for building a broader application of various types of biological molecules and easy to achieve, which might provide a promising potential for practical application in biological or clinical target analysis.

**Figure 17:**
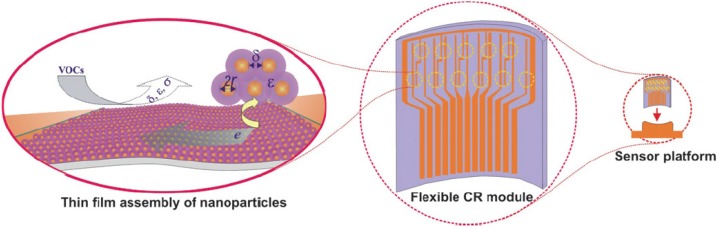
Illustrations of a chemiresistor (CR) sensor array of patterned microelectrodes on a flexible polymer substrate as a plug-and-play module, and the nanoparticle thin film assembly on the microelectrodes of the chemiresistor as sensing materials for detection of VOCs, in which the electrical properties are tuned by the nanostructural parameters (particle radius (r), interparticle distance (d or d), and interparticle dielectric medium constant (ε)). Reproduced from Wang *et al.* [140] with permission of The Royal Society of Chemistry.

There are other developments of the concept of NMNP/polymer composites for sensing applications: in this context, thin film assembly of metal nanoparticles on flexible chemiresistor (CR) arrays represents an intriguing way to address the versatility of chemical sensor design. In the Wang *et al.* work [140], thin film assemblies of gold nanoparticles in size range of 2–8 nm diameters with high monodispersity (unlinked or linked by molecular mediators) were assembled on a CR array with a polyethylene terephthalate (PET) substrate to demonstrate the flexible chemiresistor characteristics of the nanostructured materials (Figure 17). The correlation between the relative change in electrical conductivity and the change in dielectric medium constant in response to flexible wrapping of the device demonstrated the viability of manipulating the electrical responses in terms of wrapping direction. The responses of the devices to volatile organic compounds (VOCs) were analyzed in terms of particle size, interparticle properties, and substrate–film interactions. For molecularly linked films with small particle size and large interparticle spacing, which is characterized by a high percentage of organics and linker molecules, the relatively low electrical conductivity renders the change in interparticle spacing able to play a dominant role in the sensor response to VOCs with small dielectric constants. The combination of a high percentage of linker molecules in the thin film assembly and a high dielectric constant for the VOCs was found to produce a negative response characteristic. In contrast, the response characteristic for the unlinked film *via* weak interparticle interactions was dominated by the change in interparticle spacing regardless of the percentage of organics in the nanostructure. The delineation between these factors and the sensing characteristics is useful in enabling a rationale design of the nanostructures on flexible chemiresistors.

Yao *et al.* [141] describes an application of polymer encapsulated gold nanoparticles used as relative humidity (RH) sensors. The gold nanoparticles are prepared by reduction method, and the polymer, polyvinyl alcohol (PVA), is used to encapsulate Au for getting core-shell hybrid structures. Gold-polyvinyl alcohol (Au-PVA) nanoparticles were applied to construct Au-PVA a capacitive humidity sensor. By measuring the capacitance shift into different relative humidity environments, the results showed that the Au-PVA sensors had high humidity sensitivity, stability, fast humidity response and better reproducibility than the sensors prepared by uncoated nanoparticles.

#### 4.2.2. Temperature Sensors

The formation of NMNP within a polymer matrix and the change of their topological distribution induced by a variation of temperature, are the basis of the use of NMNP/polymer composites as components for temperature sensors.

**Figure 18:**
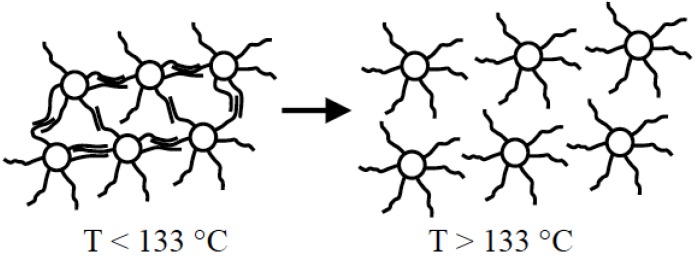
Schematic representation of the crystallization by interdigitation for nanoparticles of silver capped by dodecylthiolate. Reproduced from Carotenuto [142].

Technologically useful reversible thermochromic materials have been prepared using very simple polymer-embedded nanostructures by Carotenuto *et al.* [142]. In particular, silver nanoparticles capped by long-chain alkyl-thiolate molecules (*i.e.*, Ag_x_(SCnH2n + 1)_y_, with n > 10) spontaneously organize in aggregates because of the interdigitation phenomenon involving the linear alkyl chains bonded at surfaces of neighbouring nanoparticles (Figure 18). Owing to the alkylchain interdigitation, nanoparticles very close to each other result and an interaction among their surface plasmon resonances may take place.

**Figure 19:**
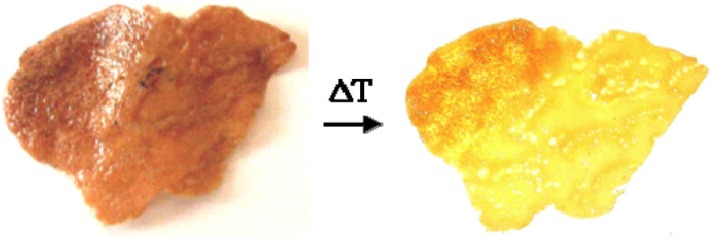
Characteristic reversible thermochromism of a film based on dodecylthiolate capped silver nanoparticles embedded into amorphous polystyrene (the material has thermally cycled for *ca*. 30 times). Reproduced from Carotenuto [142].

Surface plasmon interaction causes a splitting of the absorption band whose characteristics depend on the aggregate shape. Since shape-less aggregates are generated, a multiple-splitting of the silver surface plasmon absorption band is observed, which causes a broad absorption spreading on the whole visible spectral region. Amorphous polystyrene containing interdigitated silver nanoparticles has a dark-brown or black coloration, depending on the nanoparticle numerical density, but since the inter-particle distance slightly increases at melting point of interdigitation crystallites a reversible thermochromic effect is observed at this special temperature. In particular, the material color changes from dark-brown to yellow which is the color produced by the surface plasmon absorption of isolated silver nanoparticles (Figure 19).

This reversible thermochromism can be finely controlled by modifying the structure of thiolate groups, and precisely, the strength of interactions acting inside the interdigitation crystallites. The described thermochromic metal-polymer nanocomposites are technologically useful materials, because they can be used to measure temperature values much higher than those allowed to traditional thermochromic systems based on liquid crystals, which are usually lower than 80 °C. Consequently, these reversible thermochromic materials can be exploited in many high-temperature applications like overheating indicators, IR laser beam detectors, *etc.*

#### 4.2.3. Dichroic Response Sensors

In NMNP/polymer nanocomposite variations of the topological dispersion of the nanoparticles into the polymer matrix induced by mechanical deformations cause a significant change in color due to the different interactions of their surface plasmon resonances.

Poly(ethylene)-co-(vinyl alcohol) (EVAl)/AuNPs composites have been prepared by UV irradiation of EVAl film containing small amounts of the AuCl_4_^-^ precursor salt [129]. The formation of AuNPs is visually confirmed by film color changes from pale yellow to purple.

**Figure 20:**
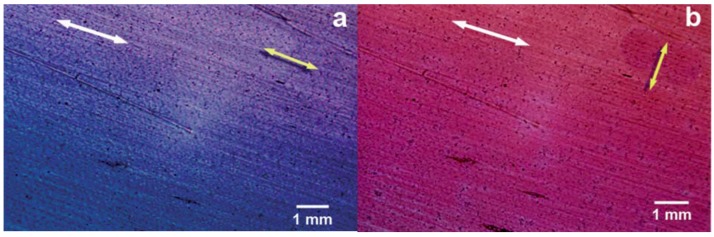
Optical microscopy images of oriented Au/EVAl44 oriented film (Dr = 5) with polarization direction of the incident light parallel (**a**) and perpendicular (**b**) to the drawing direction. The white arrows denote the stretching direction of the film, whereas the yellow ones indicate the direction of the electric vector of polarized light. Reproduced from Pucci [129] with permission of The Royal Society of Chemistry.

Uniaxial stretching of the (EVAl)/AuNPs composites promoted anisotropic packing of the embedded gold nanoparticles along the drawing direction of the film, resulting in a shift of the absorption maximum of gold well above 30–40 nm (83 nm max.) and thus producing a well-defined color change from blue to purple (Figure 20). This phenomenon may be advantageously exploited in the packaging film as a sensor for counterfeiting.

**Figure 21:**
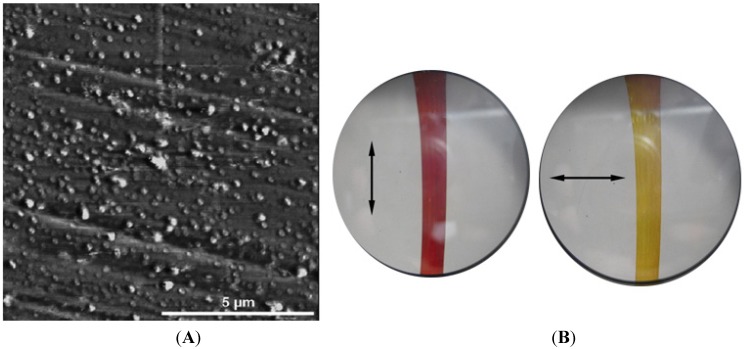
AFM image of a Ag/PVA-B120 film (**A**); Images of oriented Ag/PVA-A120 film (**B**) with polarization direction of the incident light parallel (left) and perpendicular (right) to the drawing direction. Adapted from Bernabò [110].

Recent developments have been focused in this direction in order to optimize the sensing response of AgNP/PVA nanostructured films prepared by using alternative “*in situ*” methods such as sun-(UV) or thermal promoted reduction processes [143]. The very easy and fast methods provide dispersed Ag nanoparticles (less than 4 wt%) with average diameters ranging from 15 to 150 nm depending on the type of preparation and efficiently stabilized by the chelating properties of the PVA hydroxyl groups. After uniaxial orientation, the AgNP/PVA nanocomposites show a very pronounced dichroic behavior thanks to the anisotropic distribution of the silver assemblies along the stretching direction. Indeed, oriented samples when observed through a linear polarizer show color of the films markedly depending on the relative orientation between the polarizer and the drawing direction of the film (Figure 21).

## 5. Carbon Nanotubes Thermoplastic Polymer Sensors

### 5.1. General Introduction

Carbon nanotubes (CNTs) are a dominant class of nanostructured materials that possess unique mechanical, electrical and thermal properties [144]. CNTs represent a third allotropic form of carbon and were brought to the forefront by the pioneering work of Iijima *et al.* in 1991 [145]. The exceptional properties of CNTs depend critically on their structural perfection and high aspect ratio (typically~300–1000). Single-walled CNTs (SWCNTs) consist of a single graphene sheet (monolayer of sp^2^ bonded carbon atoms) wrapped into cylindrical tubes with diameter ranging from 0.7 to 2 nm and lengths of microns. Multi-walled CNTs (MWCNTs) consist of concentric assemblies SWCNTs and are, therefore, characterized by larger average diameters. Depending on the rolling direction (chirality) of the graphene layers, different SWCNTs structures may be generated showing either metallic or semiconducting characteristics.

**Figure 22:**
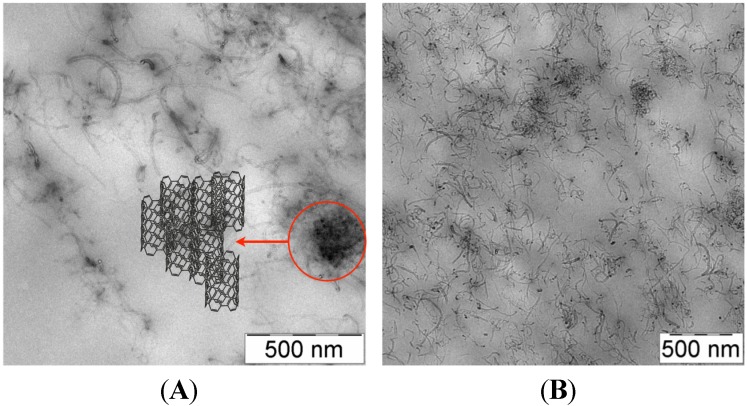
TEM micrographs of MWCNT/polymer blends. (**A**) unexfoliated MWCNT agglomerates; (**B**) an uniformly dispersed composite. Adapted from Panariello [149].

The multiple exceptional materials properties shown by both SWCNTs and MWCNTs support the virtues of their incorporation into polymeric matrices to produce nanocomposites for a variety of applications. [42,109,146] Thermoplastic polymers are attractive supporting materials for CNT since they can be easily processed and fabricated into solid-state forms such as thin films, which are often required in most sensor applications. The field of CNTs polymer composites has grown since the early seminal research of Ajayan *et al.* in 1994 [147]. CNT/polymer composites are generally described as composites with an infinite interconnected network formed by conductive fillers in an insulating matrix. According to the percolation theory, one basic assumption is that the CNT is an infinite conductor, while the polymer matrix is an infinite resistor [148]. Nevertheless, it is possible that barriers are present between conductive fillers, and the electrons need to tunnel through these barriers by quantum mechanic tunneling, which creates tunneling resistance. However, while CNTs potentially represent one of the most important filler materials for polymers, their utilization is complicated by strong van der Waals interactions between individual nanotubes that makes achieving a uniformly dispersed composite at the nanoscale difficult (Figure 22).

### 5.2. Preparation of CNT/Thermoplastic Polymer Composites

Several methods have been reported for the preparation of CNT/thermoplastic polymer composites, including solution mixing, melt-blending and in situ polymerization [150,151,152].

The solution process is at present the most effective methodology to attempt producing these nanocomposites at a small sample level. A solvent is used to dissolve firstly the CNTs, the dissolution being in general attained by ultrasonication and/or opportune amounts of surfactants in order to produce a metastable suspension of nanotubes. The polymer, swollen separately in the same solvent, is then added to the mixture. The composite is then obtained after solvent evaporation at reduced time by spin-coating the CNT/polymer suspension, thus preventing CNT re-aggregation. Another interesting approach developed to reduce the nanotube agglomeration is the hot-coagulation method in which the CNT/polymer suspension is poured into an excess of non-solvent. Ultrasonication of CNTs mixtures containing the desired polymer is the most used method for unbundling CNTs even recognizing that significant damage of their structures as well as shortening occur thus limiting the full potential of CNTs as additives in polymers. Alternative solution methods have been developed taking into account the increased solubility of nanotubes after acid functionalization. Acid-treated CNTs result highly suspendable in ethanol by ultrasonication. The addition of the mixture to a solution containing the polymer gives after further stirring and solvent evaporation the expected nanocomposite. However, besides improving nanotubes solubility in solvents, CNTs acid functionalization involves complex and time-consuming purification steps. Moreover, the acid treatment may shorten the nanotube, thus decreasing its aspect ratio, which results fundamental for the composite properties. As an alternative, sidewall covalent functionalizations including cycloaddition, such as Diels-Alder reactions, carbene addition, and nitrene addition have been reported and impart limited structural damage to the CNTs as compared with other more aggressive methods such as oxidation with nitric acid [153].

On the other hand, the melt blending process involves the dispersion of CNTs into a polymer melt by using the well-known melt-processing techniques of polymers, such as extrusion and compression molding. This procedure is particularly suitable for polymers that cannot be processed by solution techniques due to their insolubility in common solvents. High temperature and shear forces in the polymer fluid are able to break up the nanotubes bundles and the high viscosity of the melt prevents their formation during cooling. The melt blending process allows for the preparation of large-scale of CNTs/polymer mixtures but results less effective than solution blending.

The preparation process on CNTs/polymer nanocomposites by the in-situ polymerization process is generally used in the case of easily polymerizable monomers such as epoxy resins, styrene or methylmethacrylate [154]. In order to facilitate the dissolution process in the polymerization solvent or directly in the monomer, the nanotubes are either acid-functionalized or exfoliated under ultrasonication in the presence of a suitable surfactant. Notably the acid moiety covalently linked to the CNT core can be converted in a controlled radical polymerisation initiator, thus promoting the formation of polymer chain with the same length on the nanotube.

### 5.3. Vapor Sensors Based on CNT/Thermoplastic Polymer Composites

Conductive polymer composites appear to be attractive for chemical sensors development due to their good stability, lower operating temperature, good sensitivity and reproducibility to various organic chemicals, and fast response time. When a conductive CNT/polymer composite experiences an external solicitation, such as chemical vapor, strain or temperature, the conductive network will deform, and induces a change in the resistivity. This variation in the network relies on the number variation of conductive pathways, closely associated to a change of the inter-particle distance. This property gives the conductive polymer composites the potentials to be designed as sensor for various stimuli.

For example, melt-processed MWCNT/poly(lactic acid) (PLA) composites were prepared by Pötschke *et al.* [155] in order to study their liquid sensing properties on the basis of the change of electrical properties on solvent contact. The composites were prepared by melt-processing using a twin screw extruder followed by compression molding, showing electrical percolation threshold below 0.5 wt% MWNT content. Various solvents (*n*-hexane, toluene, chloroform, tetrahydrofuran, dichloromethane, ethanol, and water) were monitored in liquid immersion/drying cycles with electrical resistance variations (about 0.003–3.0 × 10^3^) dependent on the solubility parameter of the solvent that is the measure of the attractive strength between analytes and the sensing material.

However, the detection of volatile organic compounds (VOCs) appears a more appealing issue than liquid sensing. Indeed, VOCs are continuously released into the environment and some of them have adverse effects on human health. Dai *et al.* [156] reported an interesting concept for developing a new class of vapour sensors with the aforementioned characteristics. Perpendicularly aligned CNTs arrays produced by pyrolysis of iron(II) phthalocyanine were partially coated with poly(vinyl acetate) (PVAc) or polyisoprene (PI) flexible coatings by depositing a droplet of polymer solution. The aligned CNT structure confers a large well-defined surface area and the flexible layer allows the increasing of the inter-tube distance upon vapour exposure and, hence, the surface resistance across the CNT film. Notably, the as-synthesized aligned CNT arrays without the polymer coating remained unaffected when exposed to various chemical vapours. In stark contrast, a 130% increase in the resistance change was reported for an aligned CNT/PVAc composite after exposure to tetrahydrofuran vapours for several minutes. The use of PVAc/PI binary polymer coatings showed reasonably good responses also to cyclohexane and ethanol.

Another example of vapor sensing with conductive polymer nanocomposites was reported by Feller *et al.*, who investigated the sensing ability of CNT/polycarbonate (PC) transducers for toluene, methanol and water vapors [157]. Homogeneous 1 wt% CNT/PC chloroform mixtures were sprayed layer by layer onto a clean printed circuit board with an interdigitated array of copper tracks. The resistance response for the given composite was found to be coherent with the Flory–Huggins interaction parameter between solvent and polymer showing the following sensitivity ranking, toluene > methanol > water.

**Figure 23:**
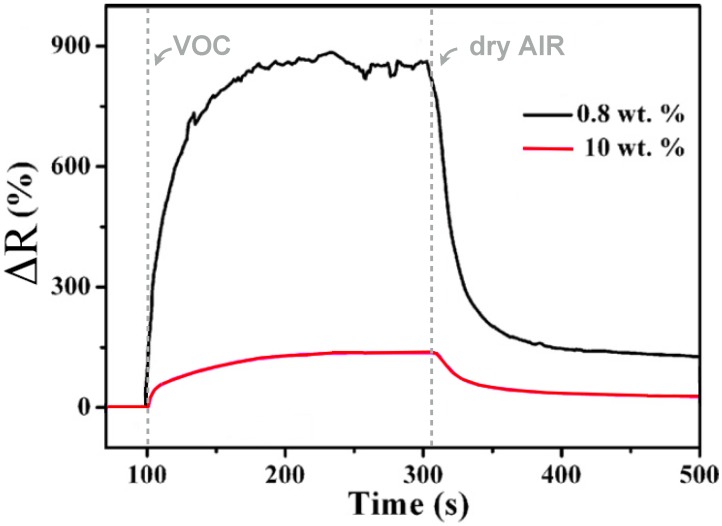
Relative resistance variations of CNT/TPU composites with different CNT contents upon exposure with 7.0 vol% of chloroform vapours. Adapted from Zhu *et al.* [158].

Zhu *et al.* have recently developed a novel kind of CNT/thermoplastic polyurethane (TPU) multifilament with VOC sensing properties [158]. The TPU multifilament was produced by melt spinning, whereas the CNT/TPU composite was prepared by immersing the TPU fibre into CNT dispersion in chloroform under sonication for 1 min. The adhesion of CNTs on the multifilament surfaces was favored by the swelling of TPU in chloroform, which even helps CNT integration within the polymer matrix during the shrinking when drying the composite. The resulting CNT/TPU composites displayed fast and reproducible electrical resistance variations upon cyclic exposure to diluted VOCs (benzene, toluene, chloroform, tetrahydrofuran, ethanol, acetone and methanol) and pure dry air (Figure 23).

The vapour responses were found to be coherent with the CNT content, vapour concentration as well as the solubility parameter of the solvents. It was proposed that the swelling effect during solvent exposure causes the disconnection of CNT networks, thus modifying the electric resistance of the sensor. For example, lower CNT loadings (0.8 wt%) resulted in larger resistance variations (about 900%), when sensing 7.0 vol% chloroform.

### 5.4. Temperature Sensors Based on Cnt/Thermoplastic Polymer Composites

**Figure 24:**
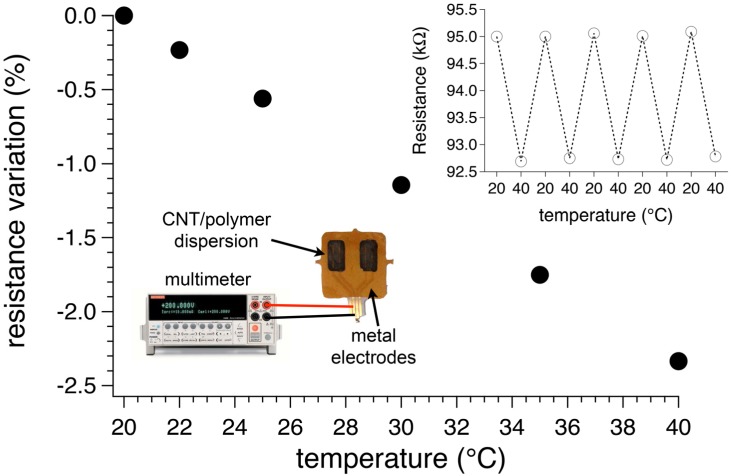
Percentage variation of resistance versus temperature for 13.2 wt% MWCNT/PVBC_Et3N nanocomposites, (inset) experimental set-up and (inset) resistance variation towards alternating heating and cooling cycles between 20 and 40 °C. Adapted from Giuliani *et al.* [164].

CNTs with either semi-conducting or metallic character show a resistivity that depends on temperature, which makes CNT nanocomposites potentially useful for the fabrication of small-size temperature sensors [159]. Both non-metallic with negative dR/dT and metallic with positive dR/dT temperature dependence of the electric resistance has been reported for single and multiwalled CNTs [160,161]. For example, Pucci *et al.* have recently investigated the dispersion of MWCNTs within poly(styrene-b-(ethylene-co-butylene)-b-styrene) (SEBS) mixtures via solution processing for the realization of miniaturized temperature sensors [162,163]. They demonstrated that solution processing via sonication induced an extensive MWCNTs degradation (average length decreased of about 40%), which affected the electrical conductivity of the nanocomposites. On the other hand, the use of alkyl-functionalized MWCNTs appeared to be more effective in preparing SEBS nanocomposites due to the higher dispersion efficiency, negligible nanotube degradation and higher electrical conductivity. The electric resistance measurements were performed on films obtained by casting the MWCNT/polymer dispersions onto a gold electrode pair supported on a polyimide film. The resulting films showed a temperature dependent resistivity with a sensitivity of −0.007 K^−1^, that is comparable to the highest values found in metals (0.0037–0.006 K^−1^), which was however partly lost after the first heating cycle up to 55–60 °C. This loss of sensitivity was attributed to the elastomeric nature of the SEBS matrix, whose mobility with temperature did not ensure a phase stability to the dispersed MWCNTs. Giuliani *et al.* proposed to overcome this issue by using as ionomeric surfactant a poly(vinylbenzyl chloride) derivative with triethylamine (PVBC_Et3N) in which 78% Cl atoms of the benzyl groups were replaced by Et_3_N [164]. They demonstrated that the effective interaction between the ionomer and the CNTs through weak van der Waals and cation-π interactions allowed the preparation of well exfoliated and undamaged nanocomposites. Moreover, the nanocomposite displayed, for MWCNT contents close to the percolation threshold, a resistance sensitivity to temperature of −0.004 K^−1^ and a very good reproducibility of the sensor response towards alternating heating and cooling cycles between 20 and 40 °C (Figure 24).

They attributed this feature to the phase stabilization of the composite provided by the high glass transition temperature (Tg) of the ionomer.

Flexible temperature sensors based on CNTs were also proposed by Sibinski *et al.* [165], that prepared a series of single yarns based on polyvinylidene fluoride fiber coated by pastes of MWCNT dispersed within poly(methyl methacrylate) (PMMA) at different concentration. They demonstrated that the system containing the overall 2 wt% of coated MWCNT showed a temperature coefficient of 0.0013 K^−1^ in a temperature range of 35–42 °C.

The flexible nature of the thermoplastic polymer hosts would also suggest the application of prepared sensors as miniaturized devices and smart textiles to be used for health and protection purposes. However, the low sensitivity of the temperature sensors is addressed to the insulating nature polymeric dispersant, which limited thermal and electronic conductivity of the final nanocomposite. This drawback could be precluded by selecting different thermoplastic surfactants bearing less insulating interaction moieties such as grafted π-conjugated aromatic groups.

### 5.5. Stress-Strain Sensors Based on CNT/Thermoplastic Polymer Composites

Thermoplastics and thermoplastic elastomers (TPE) containing CNTs have reached considerable attention in literature due to the development of stretchable resistivity-strain sensors for detecting dangerous deformations and vibrations of mechanical parts in many fields of science and engineering [166,167,168,169]. In CNT/polymer composites, applied strain induces carbon nanotube displacement/sliding on the microscale, as well as tensile deformation applied locally to individual CNT. These responses give rise to piezoresistive behaviour; that is applied tensile strains result in measurable changes in electrical resistivity across the composite length.

For example, Lanceros-Méndez investigated the piezoresisitive properties of MWCNT/triblock copolymer styrene-butadiene-styrene composites prepared by solution casting [170]. The electrical percolation threshold was less than 1 wt% for all different matrices of SBS composites and the CNT content present in the samples did not affect the high deformation capability of the polymer matrix (~1500%). Variations of the electrical resistance due to mechanical deformation were quantitatively evaluated by the gauge factor (GF, = (dR/R_0_)/(dl/l_0_), where *R* is the measured resistance and l is the length of the composite). The authors reported that 4 wt% CNT/SBS nanocomposites could be used as sensors up to 50% of deformation, with GF values higher than 100.

**Figure 25:**
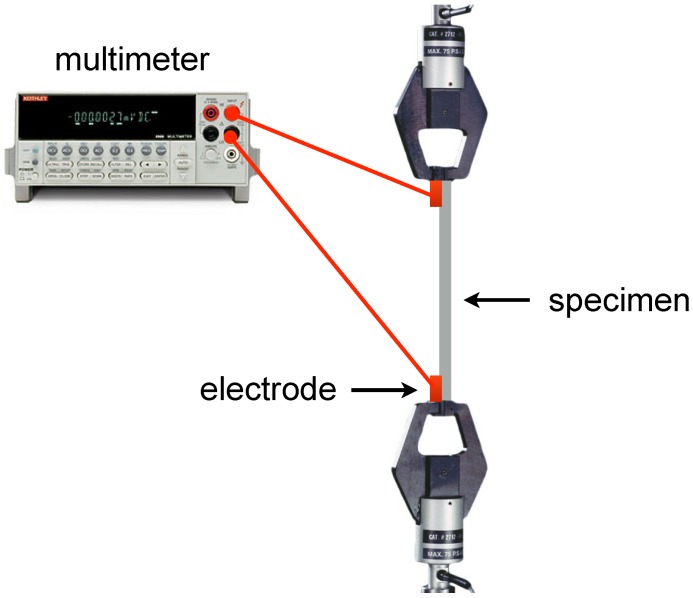
Schematic of tension test setup. Adapted from Park *et al.* [171].

Park *et al.* developed strain sensors with tailored sensitivity based on thermoplastic composites made from MWCNT/PMMA mixtures prepared by either melt processing or solution casting [171]. The nanocomposite films were obtained after solvent evaporation of compression molding of the respective mixtures. The surface resistivity of the films was correlated with the applied strains (Figure 25) and was observed to increase with increasing tensile strain.

This behavior was addressed to the reduction in conductive network density and increase in inter-tube distances induced by deformation. The highest sensitivity achieved in this study was reported to be an order of magnitude greater than conventional resistance strain gages and the sensor response of the films was reversible under cyclic loading in the elastic regime of the PMMA matrix.

PMMA, polystyrene (PS) and polycarbonate (PC) amorphous polymer matrices were efficiently used by Feller *et al.* to embed CNTs and the derived mixtures were deposited by a layer by layer procedure directly on a PET woven textile [172]. They demonstrated that adjusting the number of sprayed layers enabled to tailor both sensitivity and stability of the piezo-resistive responses in order to monitor the strain evolution in the elastic domain. The results collected support application in the field of strain sensors to monitor the deformation of a flexible, rigid and rough substrate such as a commercial boat sail.

**Figure 26:**
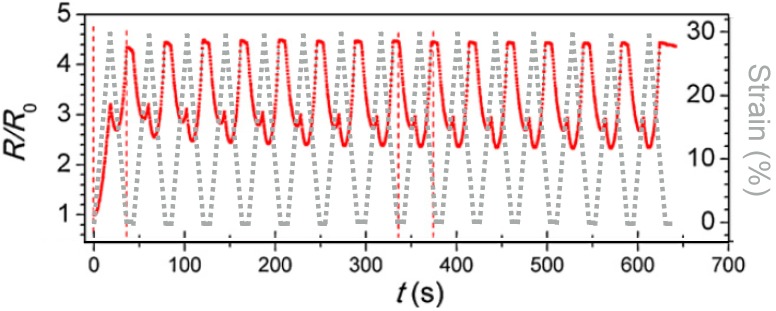
Strain sensing behaviour of coated yarn with 2 wt% of CNT upon cyclic loading at 30% strain amplitudes. Adapted from Zhang *et al.* [175].

Many examples on piezoresistive sensors are referred to some elastomers such as segmented polyurethanes that can be tailored to exploit the processing properties of thermoplastics (thermoplastic polyurethanes, TPU). The mechanical properties of TPU are due to physical interactions of the soft polyol and hard diisocyanate segments. For example, Billotti *et al.* [166] fabricated a highly conductive TPU fibres containing MWNTs and fabricated via an extrusion process. These fibres were sensitive to both static and cyclic deformation, which gave them potential uses in smart textiles with piezoresistive features. The same authors recently demonstrated that the addition of a secondary nanofiller, such as electrically conductive carbon black or an insulating needle-like nanoclay (*i.e.*, sepiolite), is able to accelerate the dynamic percolation of CNT in a polymeric matrix, in a way that is independent from its shape and electrical properties [173]. Another example reported by the same authors concerns a commercially available TPU multifilament yarn that was coated with a thermoplastic TPU/MWNT conductive polymer mixtures prepared by sonicating the components in N,N-dimethylacetamide [174,175]. Good strain sensing ability was achieved with composites already containing the 0.015 wt% CNT. In Figure 26, upon cyclic deformations at 30% strain amplitude, conductive yarns with 2 wt% of CNTs showed a reproducible positive strain effect.

This feature confers the composite material good potential as a highly sensitive fibre sensor for smart textile applications.

Recently, Bautista-Quijano *et al.* compared CNT/polyurethane composites obtained using either SWCNTs or MWCNTs after solvent evaporation of the corresponding mixtures in chloroform [176]. They found that the electrical conductivity and the piezoresistive sensitivity were higher for composites fabricated with MWCNTs than for those made with SWCNTs. The authors attributed this result on the overall metallic character of MWCNTs with respect to the semiconducting one of SWCNTs.

**Figure 27:**
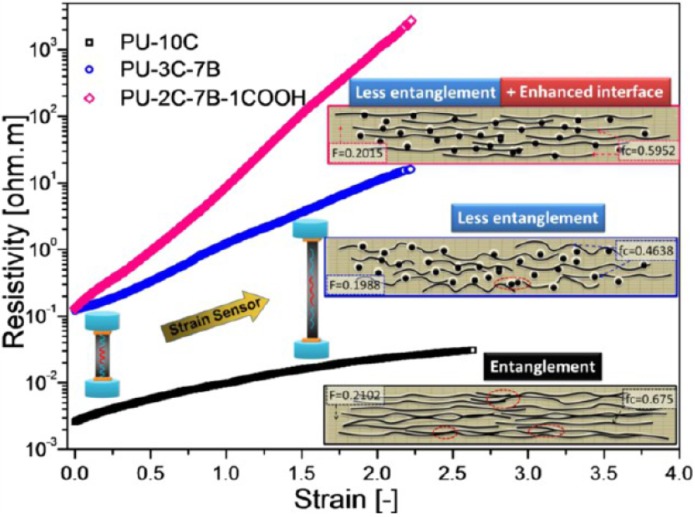
Strain-sensing behavior measurement for TPU/MWNTs/CB composites: TPU with 10 wt% of MWCNT (black curve); TPU with 3 wt% of MWCNT + 7 wt% of CB (blue curve); and TPU with 2 wt% of MWCNT + 7 wt% of CB + 1wt% of acid functionalized MWCNT (pink curve). In the inset, a possible mechanism which explains the reasons of the enhanced strain sensitivity of the composite. Copyright (2014) Royal Society of Chemistry.

In order to increase strain sensitivity, Deng *et al.* [177] reported the preparation of TPU-based strain sensors containing a mixture of carbon black (CB) and MWCNTs or acid-functionalized MWCNTs. The conductive composites were prepared by mixing tetrahydrofuran TPU solutions with the graphitic materials combined together by using 1-butyl-3-methylimidazolium bis[(trifluoromethyl) sulfonyl]imide as ionic liquid. They demonstrated that mixtures of MWNTs and carbon black could reduce the entanglement in conductive network structure, thus increasing the resistivity-strain sensitivity. Notably, the use of acid-functionalized MWNTs in the conductive composite leaded to further increase in strain sensitivity due to enhanced interfacial interaction between the conductive filler and the TPU matrix (Figure 27). This simple but effective method could allow the production of strain sensors with a large sensing capability and modulable sensitivities, that with GA ranging from 5 to more than 140,000.

The same authors have recently proposed the preparation of thermoplastic elastomer blends consisting of SBS and TPU via different melt processing procedures aimed at selectively localizing MWCNTs in the different polymer matrices [178]. According to the preparation recipe, MWCNTs can be localized preferentially within the SBS phase or the TPU segments, with which CNTs interact worse due to the higher polarity than SBS. In the first case, due to this stronger interfacial interaction, an efficient load transfer from SBS to MWCNTs occurred during material loading. In contrast, in the second case, characterized by weaker MWCNTs-TPU interactions, less efficient stress transfer occurred during stretching, thus providing much less strain sensitivity.

## 6. Conclusive Remarks and Future Perspectives

This review reports the most significant advances in the preparation and characterization of thermoplastic nanocomposites potentially exploitable as sensors, thanks to the effective dispersion of 0 D, 1 D and 2 D, even labeled, nanostructured organic or inorganic fillers. The possibility of controlling the morphology and topology development of thermoplastic-based nanocomposites, by tuning the interfacial properties of the biphase materials, has been successfully used to impart to polymer matrices new optical, conductive and thermal features, which depend on the characteristics of polymers and nanostructured substrates (often functionalized or labeled with photoresponsive organic molecules) as well as on the unique synergistic effects due to the nanoscale dispersion. 

The preparation, the inherent properties and the possible functionalization of cationic and anionic clays, noble metal nanoparticles and carbon nanotubes have been here discussed. In particular, the fillers precisely designed have been embedded in polymer matrices by using different procedures ranging from solution methodologies to in-situ polymerization approaches, self assembly technique, and melt mixing to investigate the possibility to impart sensing features.

In the case of the layered clays the target has been pursued firstly by conferring to the nanostructured inorganic system new photoluminescence characteristics by *ad hoc* modification/functionalization, generally achieved by ionic exchange, with organic molecules bearing photoresponsive chromophores or covalent bonding of the functional moieties on the layers. Particular efforts have been devoted in controlling and tailoring the nature of the aggregates and the structural arrangements of the host (the organic responsive molecule) and the guest (the clay platelets) systems to modulate or, even better, to optimize the optical properties of the clay-chromophore hybrid materials. The results collected show the feasibility of the methodologies used and even the possibility for these hybrid systems to be directly used as pH-sensors, electrochemical sensors, sensors for volatile organic compounds (VOCs) and biological molecules as well as sensors for the identification of hazardous molecules. In spite of this encouraging literature, the dispersion of functional nanoclays in a polymer matrix is however a pursued target to transfer the photophysical properties of the hybrid to the polymer matrix and then to use the optical properties as probe in deepening some effects more related in studying the morphological features (the dispersion level of the clay) than in designing polymer sensors.

Even if the authors cannot fully explain this evidence, a certain lack of synergic effects owing to the embedding of the layered functional hybrid systems in some polymer matrices, or even a detriment of the optical properties in the nanocomposite, can be supposed to affect the research strategy currently in progress. Some hybrid systems seem to work very well as sensors before the dispersion in polymer matrices, where the non-specific interaction with the polymer could actually undermine the target property. A low number of examples, although the most interesting (at least for the purpose of this review), are related to the preparation of vapour and chemical sensors and to building up a new class of photochromic devices with NLO properties. At the present, nanocomposites showing sensing properties have been prepared rather by intercalation of polyions or LBL self-assembly technique than by melt or solution methodologies thus obtaining samples at a small level. In the case of the anionic clays, for instance, the good filmability of the functional LDHs make these hybrids ready for the realization of sensor microsystems. This property has probably limited the development of thermoplastic nanocomposites prepared by dispersion of the functional LDHs in the polymer matrix or by polymerization in situ. However, future developments in this direction could lead to nanocomposites, where the control of morphology and polymer/clay interactions could likely determine new properties compared to those of the functional LDH precursors.

More mature/defined technologies seem to affect the employment of noble metal nanoparticles and carbon nanotubes in preparing thermoplastic polymer sensors. In both cases, the final, enhanced or new imparted properties of polymer nanocomposites are mainly depending on the final dimensions and alignments of aggregates/bundles stabilized by specific interactions with the polymer matrices. Molecules sensors, chemiresistors, temperature and dichroic sensors take advantage from conductivity feature and interdigitation phenomenon, depending on thermo-responsive or mechano-responsive aggregates/particles or entanglements networking. Functionalization of nanofillers is, in these cases, provided rather to better disperse the aggregates or to stabilize the clusters than to decorate the active nanostructures.

Nevertheless, there are a number of challenges to be addressed to fulfill the application of thermoplastic polymer/carbon nanotube composites for sensing applications, being the elevated cost of high purity CNTs and their scalable controlled dispersions still open issues. One accessible solution would be the use of tailored functionalized thermoplastic polymers whose functional and less insulating moieties would help in carbon nanotubes exfoliation while providing higher sensor sensitivity for less amount of conducting filler. If all these drawbacks are properly addresses, these polymer nanocomposites will have a striking impact in the future of sensors based on carbon nanomaterials.
